# Topography and Ensemble Activity in the Auditory Cortex of a Mouse Model of Fragile X Syndrome

**DOI:** 10.1523/ENEURO.0396-23.2024

**Published:** 2024-05-07

**Authors:** Simon L. Wadle, Tamara C. Ritter, Tatjana T. X. Wadle, Jan J. Hirtz

**Affiliations:** Physiology of Neuronal Networks, Department of Biology, RPTU University of Kaiserslautern-Landau, Kaiserslautern D-67663, Germany

**Keywords:** auditory cortex, fragile X syndrome, two-photon imaging

## Abstract

Autism spectrum disorder (ASD) is often associated with social communication impairments and specific sound processing deficits, for example, problems in following speech in noisy environments. To investigate underlying neuronal processing defects located in the auditory cortex (AC), we performed two-photon Ca^2+^ imaging in *FMR1* (*fragile X messenger ribonucleoprotein* *1*) knock-out (KO) mice, a model for fragile X syndrome (FXS), the most common cause of hereditary ASD in humans. For primary AC (A1) and the anterior auditory field (AAF), topographic frequency representation was less ordered compared with control animals. We additionally analyzed ensemble AC activity in response to various sounds and found subfield-specific differences. In A1, ensemble correlations were lower in general, while in secondary AC (A2), correlations were higher in response to complex sounds, but not to pure tones. Furthermore, sound specificity of ensemble activity was decreased in AAF. Repeating these experiments 1 week later revealed no major differences regarding representational drift. Nevertheless, we found subfield- and genotype-specific changes in ensemble correlation values between the two times points, hinting at alterations in network stability in *FMR1* KO mice. These detailed insights into AC network activity and topography in *FMR1* KO mice add to the understanding of auditory processing defects in FXS.

## Significance Statement

Communicative challenges often observed in people with autism spectrum disorder might be due to defects in cortical brain circuits responsible for sound analysis. To investigate these in detail, we used a mouse model of fragile X syndrome (FXS), which often is associated with autism spectrum disorder in humans. We found several alterations compared with control animals, including a less well-ordered topography of frequency analysis in the auditory cortex. Furthermore, neuronal population activity patterns in response to various sounds were altered. This was also highly dependent on whether pure tones or complex sounds were presented. These data help to understand the causes of sound processing defects in FXS.

## Introduction

Autism spectrum disorder (ASD) patients display persistent impairments in social communication and social interactions accompanied by restricted, repetitive behavioral patterns at various severity combined with increased sensitivity to sensory stimuli (American Psychiatric Association, 2013). Auditory deficits are prevalent in ASD, most often described as impaired auditory filtering and difficulties of understanding speech in noise (Alcántara et al., 2004; Tomchek and Dunn, 2007; Ashburner et al., 2008; Groen et al., 2009; DePape et al., 2012; Schafer et al., 2013; Schelinski and von Kriegstein, 2020). Loss-of-function mutations in the *FMR1* gene leading to fragile X syndrome (FXS) are the most frequent monogenetic cause (6–8%) of ASD in humans (Muhle et al., 2004; Fyke and Velinov, 2021), with a general prevalence of ASD at ∼2% of children (Baio et al., 2018). Furthermore, various forms of ASD cause misexpression or malfunction of fragile X messenger ribonucleoprotein (FMRP; Assaf et al., 2010; Just et al., 2012). Therefore, *FMR1* knock-out (KO) mice have been used frequently as a mouse model of ASD to investigate deficits in neuronal processing caused by altered expression of the FMRP encoded by the *FMR1* gene locus. Loss of FMRP causes misregulations in translational processes and alterations in direct protein interaction partners of FMRP (Bülow et al., 2022). At the synapse, these functions include indirect and direct control of presynaptic Ca_V_ activity by activating BK_Ca_ channels or directly interacting with Ca_V_ (Deng et al., 2013; Ferron, 2016) and translation and localization of diacylglycerol lipase-α which uses the endocannabinoid pathway at the postsynaptic side, again influencing presynaptic Ca_V_ activity, leading to increased transmitter release (Jung et al., 2012; Fyke and Velinov, 2021). Furthermore, in the cortex of *FMR1* KO mice, misregulation of the matrix metalloprotease-9 (MMP-9), caused by a lack of FMRP, leads to decreased perineuronal net formation around parvalbumin (PV) interneurons, altogether disturbing E/I balance and leading to hyperexcitability (Gibson et al., 2008; Contractor et al., 2015; Wen et al., 2018).

At the level of the auditory brainstem, a wide array of alterations have been reported in ASD patients and *FMR1* KO rodents, including disrupted branching and orientation of dendritic trees (Kulesza and Mangunay, 2008), decreased neuron size (Ruby et al., 2015), hyperexcitability (El-Hassar et al., 2019), altered expression of K_V_3.1 (Strumbos et al., 2010), altered synaptic transmission and connectivity (Garcia-Pino et al., 2017; McCullagh et al., 2017; Curry et al., 2018), and decreased frequency selectivity (Garcia-Pino et al., 2017). These studies are important contributions to understanding auditory defects in ASD, as this disorder disrupts auditory perception in the temporal envelope (Robertson and Baron-Cohen, 2017), which is highly dependent on fast and precise synaptic transmission in the auditory brainstem. However, deletion of FMRP exclusively in the forebrain of mice also causes some of the *FMR1* KO phenotypes, suggesting that local cortical circuitry is directly affected by genetic causes of ASD (Lovelace et al., 2019). Moreover, *FMR1* KO mice display impaired auditory cortical plasticity (Kim et al., 2013; S. Yang et al., 2014) and impaired auditory-related learning (Zhao et al., 2005; Reinhard et al., 2019). In the auditory cortex (AC) of FXS patients and *FMR1* KO mice, electroencephalogram studies revealed increased amplitudes in event-related potentials and reduced habituation to repeated sounds, indicating a higher noise level in AC activity (Castrén et al., 2003; Ethridge et al., 2016; Lovelace et al., 2018; Wen et al., 2019). On the single-unit level, AC neurons in *FMR1* KO mice exhibit stronger responses to brief tones, broader frequency tuning, and reduced frequency modulation selectivity (Rotschafer and Razak, 2013), again indicating spectrotemporal processing deficits. However, very little is known about cortical network activity alterations at the cellular level in *FMR1* KO or ASD.

We here performed an in-depth study of sound-evoked activity patterns within the AC of *FMR1* KO mice using two-photon Ca^2+^ imaging to maintain single-cell resolution while observing hundreds of neurons within one experiment. We reveal decreased bandwidth (BW) of neurons, altered topography of frequency-related activity, and alterations in ensemble activity in response to simple and complex sounds. Our results contribute to the understanding of altered AC activity patterns in *FMR1* KO mice that might underlie impaired sound processing in FXS.

## Materials and Methods

### Animals

Male hemizygous *FMR1* KO mice were generated by inserting the homologous recombination target vector pMG5 into exon 5 of the *FMR1* gene, resulting in a loss of FMRP (Bakker et al., 1994), and obtained from Jackson Laboratories (B6.129P2-Fmr1tm1Cgr/J, Strain #:003025). They were bred in the animal facility of the RPTU University of Kaiserslautern-Landau with animals of the same genetic background to obtain heterozygous females. These were bred either with KO males to obtain more hemizygous KO males and wild-type (WT) males, as well as homozygous KO females, or with WT males to also obtain WT females. Food and water were provided *ad libitum*, and all animals were group housed at a 12 h light/dark cycle. Genotyping was carried out on a material left over from ear punch markings. The study was performed in accordance with the guidelines of the German Animal Welfare Act and the European Directive 2010/63/EU for the protection of animals used for scientific purposes. Animal experiments were approved by the regional council of Rhineland-Palatinate (Landesuntersuchungsamt Rheinland-Pfalz) under the file numbers G15-2-076, G19-2-032, and G21-2-072. For in vivo experiments, surgical procedures started at Postnatal Day (P)32, and succeeding imaging experiments were performed up to P70. Slice experiments were conducted between P50 and P70. Animals of both sexes were used (WT, 4× female, 4× male; KO, 3× female, 2× male), with data pooled.

### Injection of viral vectors, habituation, and window implantation

In order to provide sufficient analgesia, mice were injected with carprofen (Rimadyl®, Zoetis; 5 mg/kg body weight) intraperitoneally 30 min prior to the initial anesthesia with isoflurane (3–5%, Fluovac system, Harvard Apparatus). The anesthetized mouse was placed on a heating mat (TC-1000 Temperature Controller, CWE) which maintained the body temperature monitored through a rectal thermometer and fixed in a stereotactic frame (Model 900, David Kopf Instruments). The head holder was connected to the Fluovac system which allowed continuous supply of isoflurane (1–3%) during the whole operation. The level of anesthesia was checked regularly by the paw withdrawal reflex, triggered by an intertoe pinch, and isoflurane concentration was adjusted accordingly. Drying-out of the mouse's eyes was prevented by applying an eye ointment. After the fur was shaved from the scalp, Braunol® (B. Braun Melsungen) was applied for disinfection purposes, and 50 µl of lidocaine (Lidocainhydrochlorid 20 mg/ml, bela-pharm) was injected subcutaneously for local analgesia. After 5 min waiting time, the scalp was opened along the midline and removed over the right hemisphere. Afterward, the head was tilted by 45° to the right, and the skin over the left hemisphere was pushed aside to reveal the underlying skull and muscles. The musculus temporalis was then partly removed to access the skull area over the AC. The area of the AC was then approximated by topographic structures, and two injection sites were chosen. A small hole was drilled with a dental drill. The tip of a syringe (NanoFil, World Precision Instruments) containing 3.8 × 10^12^ GC/ml of the adeno-associated viral vector (AAV)1-hSyn-jGCaMP7f (pGP-AAV-syn-jGCaMP7f-WPRE was a gift from Douglas Kim and GENIE project (Addgene viral prep #104488-AAV1)) which was inserted through the hole. Seven hundred fifty nanoliters of the vector solution was injected at a rate of 80 nl/min at 500 µm depth. After successful injection, the syringe was kept in place for 5 min, and the procedure was subsequently repeated for the second injection site. A titanium anchor was attached using dental cement (C&B Metabond; Parkell). The remaining skin from the left side of the scalp was then again pulled above the injection sites and cemented to the head plate, sealing the operation site. The animal was then retracted from the stereotactic frame, placed on a heating mat until fully awake, and then brought to its home cage for recovery. To prevent dehydration during the operation, 0.5 ml of 0.9% NaCl solution was administered subcutaneously 60 min after the start of the surgery. For analgesia and to prevent inflammation, carprofen (5 mg/kg body weight) was administered for 2 subsequent days. The well-being of the animal was monitored daily.

Before performing imaging experiments in awake mice, animals were first habituated to the experimental situation, starting earliest 3 d after AAV injection. In each session, animals were brought to a treadmill (LN treadmill, Luigs & Neumann) under the imaging setup and were allowed to freely explore their surroundings for 15 min. Subsequent head fixation lasted initially for 10 min and increased by 25–30 min each day until 2 h were reached, resembling the maximal time for one imaging session. Animals were not habituated on the day of window implantation surgery and on the 2 following days. After five habituation sessions, none of the animals showed any signs of stress, for example, cowering, clinging, and sudden fast running, and were therefore used for subsequent imaging experiments.

Eight days after AAV injection, a window was implanted into the skull, following the general surgical procedures described above. The joint between the cement and remaining skin over the left hemisphere was reopened. The boundary of a round piece of the skull, ∼3 mm in diameter, over the AC was thinned down by tracing the edge with a dental drill. Once the remaining skull encircled by the furrow was loose, it was removed with a fine kinked probe. The dura mater was removed with a small needle and fine forceps. A stack of 2 × 3 mm round cover glass (thickness #0, Warner Instruments) with 1 × 4 mm round cover glass (thickness #1), glued together by a UV-curing adhesive (NOA 60, Norland Optics), was lowered onto the brain, sealing the opening in the skull. The stack was then fixed with dental cement. The remaining exposed tissue and skull were sealed with dental cement as well. The animal was then retracted from the stereotactic frame, placed on a heating mat until fully awake, and then brought to its home cage for recovery. To prevent dehydration during the operation, 0.5 ml of 0.9% NaCl solution was administered subcutaneously 60 min after the start of the surgery. For analgesia and to prevent inflammation, carprofen (5 mg/kg body weight) was administered for 2 subsequent days. The well-being of the animal was monitored daily.

### Sound stimulation

The light box as well as the microscope and treadmill position was covered with a sound-attenuating foam (Basotect®, BASF) protecting the recording site from external background noise as well as scanner noise. With this, the sound pressure level (SPL) of ambient noise in the relevant hearing range for mice (1–90 kHz) and in the range of frequencies used for sound stimulation (4–100 kHz) approximated 35 dB SPL and 20 dB SPL at max, respectively. Ambient noise was recorded with a high-sensitive microphone (378A06, 3–40,000 Hz, 12.6 mV/Pa, inherent noise: 22 dB(A) re 20 µPa, PCB Piezotronics), and the corresponding signal was amplified by an analog amplifier [MA3, Tucker-Davis Technologies (TDT)], coupled with an analog-to-digital multifunction processor (RX6, TDT) controlled by SigCalRP (v4.2, TDT). Daily calibration of the speaker was done using the same equipment except for a less sensitive microphone, which could record higher frequencies up to 100 kHz (Model 378C01, 4–100,000 Hz, 2.01 mV/Pa, inherent noise: 42 dB(A) re 20 µPa, PCB). The SPL during tone presentation (4–100 kHz) was recorded and used to adjust driving voltages of the speaker for each frequency according to the desired SPL. These normalized values were then exported to MATLAB, and filter coefficients were calculated to be used for the online finite impulse response filter of sound presentation during experiments. During two-photon imaging, two groups of acoustic stimuli were presented. Group 1 consisted of 17 different pure tones (PTs) (4–64 kHz, four equivalent steps per octave; 250 ms tone length followed by a 1 s pause), AM tones (same carrier frequencies and time course as PTs, 20 and 40 Hz modulation frequency, 70 dB SPL), and complex acoustic stimulations, consisting of mouse vocalization mimic (3.8 kHz fundamental frequency and second and third harmonic), AM mouse vocalization mimic (same as vocalization mimics but 1 kHz modulation frequency), 12 natural animal vocalizations (1 s pause between vocalizations, 70 dB SPL; all vocalizations downloaded from http://www.avisoft.com/animal-sounds/), and an overlay of all 12 natural animal vocalizations. We thank Matthias Göttsche (Stocksee, Germany) for allowing us to use the recordings of the Blasius's horseshoe bat. To provide enough data for a suitable analysis, only vocalizations with a duration of ≥0.55 s were chosen for analysis, that is, 10 vocalizations. They were randomly presented within their sound group (PTs, AM tones, vocalization mimics, or natural animal vocalizations) during each of the 10 repetitions. Group 2 was used to create frequency response areas (FRAs). Therefore, PT sequences were presented with five different SPLs (30–70 dB SPL increasing in 10 dB steps if each tone/vocalization was high-pass filtered at 4 kHz). For widefield imaging, five PTs (4, 8, 16, 32, 64 kHz, 500 ms tone; 5 s pause between tones) were randomly presented during each of the 16 repetitions and played at 50, 60, and 70 dB SPL. All stimuli were created with MATLAB 2020a (MathWorks) controlling a script written in RPvdsEX (v88, TDT) and loaded to the RX6 digital-to-analog converter. The output signal of the RX6 was passed by an electrostatic speaker driver (ED1, TDT) and finally transformed into acoustic signals by an electrostatic free field speaker (ES1, TDT), positioned ∼10 cm from the ear of the mouse contralateral to the window.

### In vivo Ca^2+^ imaging

For awake in vivo Ca^2+^ imaging, the cranial window was covered with an ultrasound gel (Anagel, Ana Wiz) for recordings with a 10× water immersion objective (IMPPLFLN, Olympus K.K.) and 16× water immersion objective (CFI75 LWD, 0.8 NA, Nikon). All recordings were obtained using the Ultima Investigator microscope (Bruker AXS SAS) with the objective tilted by 45°, maintaining the animal in an upright position. During all recordings, the animal was awake and able to move on a treadmill but was fixed with the head anchor. As an excitation source for two-photon imaging, a Chameleon Vision II Titan:Sapphire laser (Coherent), controlled by Chameleon Vision (v2.83, Coherent), with 140 fs pulse duration and 80 MHz repetition rate, with built-in precompensation, was used, tuned to 940 nm. The laser intensity was adjusted with a Pockels cell (Model 302RM Driver, Conoptics) and was in the range between 13 and 65 mW. Imaging was performed using a Galvo-Resonant 8 kHz scanner, recording a 512 pixel × 512 pixel field of view (FOV), covering ∼820 µm × 820 µm, at 29.76 frames/s. The emitted fluorescence was collected by the 16× objective and guided through a green emission bandpass filter onto a GaAsP photomultiplier tube (Bruker). All imaging components were controlled by Prairie View (v5.5.64.500, Bruker), and parameters were set by MATLAB 2020a via Prairie Link (v5.5.0.48, Bruker). During all recordings, the movement of the mouse on the treadmill was registered by two hall sensors.

On the first day of imaging, the FOVs were chosen to cover as much area as possible of the cranial window, depending on the area of transfected tissue. The first FOV was chosen as the origin of a coordinate system, and coordinates of each FOV were saved and together with a reference image used for guidance on the subsequent days. During Day 1, PTs, AM tones, and complex sounds were presented at 70 dB SPL. On the next day, coordinates and vasculature were used to find the same FOVs on a coarse scale. On a finer scale, the same position was searched by accurately matching pixel coordinates of cells from the live image with the reference image and scrolling on the *z*-axis until most cells from Day 1 were detectable. During imaging on this day, PTs with different SPLs (30–70 dB) were presented. To increase the number of neurons per subfield, on Days 4 and 5 of imaging, the same *XY*-coordinates per FOV were used, but a different depth was chosen. It was assured that no cells from the first depth were visible, optimizing the increase in the number of cells. The resulting depths ranged from 180 to 270 µm. The sound stimulation paradigm on Days 4 and 5 was identical to Days 1 and 2, respectively. The same imaging pattern was then repeated 7 ± 1 d later for most FOVs and depths. In some cases, recordings could only be performed in the first week, due to the worsening of the optical conditions of the window. In cases of statistical comparison of datasets across the 2 weeks, only FOVs recorded in both weeks were included. However, analysis was not limited to neurons only visible/active on both experimental days. At the end of each session, the animal was brought back to its home cage.

For widefield imaging, performed on Day 3, illumination with blue light was achieved by a LED (470 nm, Thorlabs). The emitted fluorescence was passed through a green emission filter onto a scientific CMOS camera (Prime 95B, Teledyne Photometrics), controlled by Micro-Manager (v2.0, “https://micro-manager.org”). The corresponding FOV size covered ∼1.5 mm × 1.5 mm, resulting in a 1,024 pixel × 1,024 pixel image. Data were collected, after focusing roughly 200 µm below the pial surface, at a rate of 10 Hz with an exposure time of 60 ms.

### Analysis of in vivo data

For analysis of widefield imaging data, the procedure of image processing was adapted from Romero et al. (2019). Raw images were downsampled to a 256 pixel × 256 pixel resolution. Small drifts in fluorescence signal were removed by computing a temporal baseline (F0) for each pixel from a polynomial fit (Degree 3) of a 15 s sliding window (Chronux toolbox, MATLAB). The change in fluorescence was calculated for each frame as percent change from the temporally smoothed signal (Δ*F*/*F*·100). These amplitudes were used for further analysis. Baseline activity levels for each stimulus were defined for each pixel by creating a histogram of amplitudes of all frames during the 2 s prestimulus period. To check for tone-evoked responses, the maximum amplitude was picked from the 750 ms period after tone onset and averaged with the preceding and following frame. In cases where the resulting value exceeded the prestimulus baseline activity distribution by at least two standard deviations (*z*-score >2), the response was characterized as tone evoked. A frequency-specific response amplitude was only calculated when a tone-evoked response occurred in a minimum of 4 of 16 repetitions. The frequency-specific response was then calculated as the mean of all significant tone-evoked response amplitudes to the respective frequency. The frequency eliciting the highest mean response amplitude within a pixel was set as the best frequency (BF) of that given pixel. As one FOV acquired with the 10× objective covered only a part of the AC, multiple overlapping FOVs were necessary in order to create a gapless BF map. The frequency-specific response amplitudes of each pixel within a FOV were normalized to provide comparability. In cases where one pixel was represented more than once (due to overlapping FOVs), the BF with the higher normalized mean response amplitude was chosen. Next, a vector-based calculation of reversal points, similar to the analysis in Romero et al. (2019), was provided as follows to assist subfield parcellation. First, centers of existing low-frequency hubs were identified. From each of these, a set of 1,440 radial vectors from 0 to 360° (0.25° step size) were drawn. The mean BFs along each radial vector (±1°) were smoothed with a moving average (window size 10 frames). The smoothed values were then fitted with a Gaussian filter (Degree 3), so that reversal points (first maxima) could be marked in the BF map. The end of the AC was defined as the point, where 10 pixels in a row showed no sound-evoked response at all. The marked reversal and end points within the BF map served as a template for the “drawassist” function of MATLAB. Thereby, the subfield borders could be drawn by hand, but the outline was corrected by the information of the underlying BF map. Assignment of A1, AAF, and A2 was performed, based on existing knowledge from earlier studies (Tsukano et al., 2015, 2016; Romero et al., 2019).

For two-photon data, recordings were processed with suite2p (https://suite2p.readthedocs.io/; Pachitariu et al., 2017), first correcting for rigid as well as nonrigid movement shifts. Next, region of interest (ROI) detection, signal extraction, and local neuropil signal extraction were carried out. ROI fluorescence traces, subtracted by 0.7 times neuropil traces, were then deconvolved using the OASIS algorithm (Friedrich et al., 2017), and the resulting spiking probabilities were used for most of later analyses. ROIs were grouped as “cell” or “noncell” by a trained classifier depending on the activity parameter and the parameter of the ROI shape. This automatic classification of ROIs as cells or noncells was manually reviewed. The data were then exported to MATLAB for further processing. To rescale ROI fluorescence traces and neuropil traces, first a wavelet denoising was carried out by utilizing the MATLAB function “wdenoise” (Wavelet Toolbox, MATLAB 2022a). Denoised traces were then scaled by 0.86, and the subtracted noise was added again. Deconvolution was performed by the OASIS algorithm, and following analyses were identical to the unscaled trace analyses.

AC activity is influenced during movement by inhibiting neuronal activity (Nelson et al., 2013). Therefore, phases of running which exceeded 1 cm/s were excluded from the activity traces for analysis. Furthermore, unresponsive neurons were removed from analysis if their peak signal-to-noise ratio (Eq. 1) was below 36 dB for the whole activity trace:

(1) $$\mathrm{PSNR}=20\;*\;\mathrm{lo}g_{10}\left(\frac{\max(F_{\mathrm{raw}}-F_{n})}{\sigma_{n}}\right)$$

where *F*_raw_, *F_n_*, and *σ_n_* as the ROI trace, the neuropil trace, and the standard deviation of the neuropil trace, respectively.

Neurons were defined as PT responsive by comparing the mean spiking probabilities 400 ms prestimulus and 400 ms poststimulus onset. A one-way ANOVA compared both distributions for each frequency-intensity distribution, and if *p* < 0.01 in at least one PT-SPL combination, neurons were classified as PT responsive, and others were excluded. In case of the animal running during sound stimulations, the given repetition was removed from analysis, and the complete FOV was disregarded for analysis in case fewer than five repetitions without running were recorded. Mean poststimulus responses of each frequency were averaged across SPLs, resulting in a single mean value per frequency. These points were then fitted with a unimodal and bimodal Gaussian (Eqs. 2 and 3, respectively) fit function to check for single- or double-peak tuning, respectively:

(2) $$\mathrm{unimodal}\;\mathrm{gauss}=\;A_{1}\;*\;e^{-{\left(\frac{x-B_{1}}{C_{1}}\right)}^{2}}+D$$

(3) $$\mathrm{bimodal}\;\mathrm{gauss}=\;A_{1}\;*\;e^{-{\left(\frac{x-B_{1}}{C_{1}}\right)}^{2}}+A_{2}\;*\;e^{-{\left(\frac{x-B_{2}}{C_{2}}\right)}^{2}}+D$$

To adjust for the different number of parameters of the two fits, the adjusted coefficient of determination (*R*^2^adj) was used to decide which fit was more suitable. If both fits resulted in *R*^2^adj < 0.4, the neuron was classified as “irregular tuned”; otherwise, the fit that resulted in a higher *R*^2^adj was used to assign a “single-” or “double-peak tuning”. Furthermore, the tuning BW of each peak was determined as the full width at half maximum (Eq. 4):

(4) $$\mathrm{BW}=2\;*\;\sqrt{2\;*\;\log_{10}(2)\;*\;\frac{C_{1/2}}{\sqrt{2}}}$$

In each FRA, the frequency that elicited the highest response was defined as the BF, regardless of SPL. Next, local FOV coordinates from all neurons were converted into a global coordinate system. Global coordinates from each neuron were used to calculate the local BF distribution. For each neuron, its BF and the BFs of all neurons within 100 µm were extracted. Then, the interquartile range (IQR) of the distribution was calculated as a measure of local heterogeneity. If less than five neurons were within 100 µm radius (including the center neuron), no IQR was calculated.

To analyze which sounds evoke activity in different ensembles of neurons, a correlation analysis was performed related to Bathellier et al. (2012). Hence, the activity of each neuron in a time window of 0.4 s (0.55 s in case only complex sounds were analyzed) from the start of acoustic stimulation was averaged, and the resulting values were combined to a “cell vector”. Vectors were then correlated across sounds (using Pearson’s correlation), determining the similarity, and these correlation values were averaged across repetitions, describing the reliability of responses. As for FRA analysis described above, in case of the animal running during sound stimulations, the given repetition was removed from analysis, and the complete FOV was disregarded for analysis in case fewer than five repetitions without running were recorded (for details see above). The hierarchical ordering of the resulting correlation matrix was carried out using the unweighted average distance. The correlation matrix was exported in R (R Language, v4.1.2, https://www.*R*-project.org/) where clusters were defined by a hierarchical cluster tree using dynamic tree cut (Langfelder et al., 2007, method “hybrid”, “deepsplit” set to 2.5). To determine the similarity of cluster content between 2 experimental days 1 week apart, for each FOV, the content of a given cluster observed in Week 1 was compared with the content of all clusters for Week 2, and the cluster with the most similar content was chosen as the counterpart. The fraction of sounds shared was then averaged across all clusters observed in Week 1 to determine one similarity value for the FOV. For neuron correlations across the week, the two cell vectors described above were correlated for each given sound and repetition (but not between sounds), and the values were averaged for one FOV.

### Code accessibility

The code/software described in the paper is freely available online at https://github.com/HirtzLab/Imaging_auditory_cortex_fmr1_KO. The code is available as Extended Data. In the present study, the code was run on standard PCs using Windows 10 operating systems.

10.1523/ENEURO.0396-23.2024.d1 Extended Data Code used in the present study. See comments at beginning of files for details. Download Extended Data, ZIP file.

### Acute slice physiology

Animals were anesthetized with isoflurane (5%) and subsequently decapitated. The head was immediately submerged in an ice-cold NMDG preparation solution (in mM: 93 NMDG, 30 NaHCO_3_, 20 HEPES, 25 d(+)-glucose, 3 myo-inositol, 2.5 KCl, 3 Na-pyruvate, 0.5 CaCl_2_, 10 MgCl_2_, 1.2 NaH_2_PO_4_, 5 ascorbic acid), pH adjusted to 7.4 using HCl, bubbled with carbogen (5% CO_2_/95% O_2_). The brain region containing the AC was cut out and removed from the skull. About 270-µm-thick coronal slices were prepared using a vibratome (Leica VT 1200S, Leica) containing the ice-cold NMDG-preparation solution. For recovery, slices were then transferred to a beaker containing 37°C NMDG preparation solution and after 11 min incubation time stored at room temperature in artificial cerebral spinal fluid (aCSF; in mM: 125 NaCl, 25 NaHCO_3_, 10 d(+)-glucose, 3 myo-inositol, 2.5 KCl, 2 Na-pyruvate, 2 CaCl_2_, 1 MgCl_2_, 1.25 NaH_2_PO_4_, 0.44 ascorbic acid), pH 7.4, bubbled with carbogen until they were used for electrophysiological experiments.

Electrophysiological recordings, accompanied by single-cell Ca^2+^ imaging, were performed on an electrophysiological rig. Acute brain slices containing the AC were transferred into a recording chamber and fixed with a U-shaped platinum–iridium grid stringed with nylon strands. The chamber was then mounted on an upright microscope (Eclipse E600FN, Nikon), equipped with differential interference contrast optics, appropriate objectives (Nikon 4× CFI Achromat, 0.1 NA; Nikon 60× CFI Fluor W, 1.0 NA), and a scientific CMOS camera (Iris 9, Teledyne Photometrics), controlled by Micro-Manager (v2.0). During imaging experiments, a blue light LED (470 nm, Thorlabs) was used to illuminate the whole slice (1.1 mW/cm^2^ at maximum), and the emitted fluorescence was guided through a bandpass filter (500–550 nm) and captured by the camera. Once transferred to the microscope, the slices were continuously perfused with aCSF (room temperature, pH 7.4, bubbled with carbogen) using a peristaltic pump (ISM796B, Ismatec). Pipettes pulled from borosilicate glass capillaries with a filament (GB150F-8P, Science Products) using a horizontal puller (Flaming Brown Micropipette Puller P-87, Sutter Instruments) had resistances ranging from 2.5 to 4 MΩ when filled with an internal solution [in mM: 140 K-gluconate, 10 HEPES, 1 MgCl_2_, 2 ATP-Na_2_, 0.3 GTP-Na_2_, 0.05 Oregon green BAPTA-1 (OGB-1)].

Whole-cell recordings were obtained using a patch-clamp amplifier (EPC9, HEKA Electronics) and a micromanipulator (SM-I, Luigs & Neumann) linked to the head stage. Capacitive transients were neutralized, and series resistance was compensated by 50–70%. The liquid junction potential (15.4 mV) was corrected online. During the 10 min filling time of the cell with the internal solution, parameters for AP generation, that is, intensity and duration of the injected current, were determined. Injected currents ranged from 0.5 to 0.8 nA with a duration of 2–8 ms. Once a suitable concentration of OGB-1 was achieved, rectangular current injections were performed at different frequencies while simultaneously recording fluorescent responses. The protocols were repeated up to three times. The obtained electrophysiological data were digitized with a sampling rate of 50 kHz and low-pass filtered at 8.3 kHz. Imaging data were sampled at 40 Hz with an exposure time of 25 ms. Image sequences recorded from OGB-1-filled cells were analyzed using a custom-written MATLAB GUI allowing to display electrophysiological and fluorescence traces in parallel. Baseline for Δ*F*/*F* values was calculated by taking the mean of 75 ms prior to the peak.

Data were analyzed using a custom-written MATLAB (MathWorks) code with the HEKA Patchmaster Importer (Keine, 2019). To normalize changes in fluorescence across animals and slices, Δ*F*/*F* values were calculated by averaging fluorescence 330 ms prior to each peak yielding a local baseline, which was used to normalize the corresponding peak. Hence, Δ*F*/*F* values for each stimulation intensity could be obtained.

### Data visualization and statistics

Bar graphs and values in text present mean ± standard error of mean with number in bar depicting *n*-number. Normal distribution was tested with Kolmogorov–Smirnov test. Normally distributed datasets were compared using paired or unpaired, two-tailed *t* tests. Distribution-free datasets were compared using Wilcoxon signed rank tests or Mann–Whitney *U* tests for paired or unpaired datasets, respectively. Significance levels are as follows: *p* < 0.05 *, *p* < 0.01 ** and *p* < 0.001 ***.

## Results

**Figure 1.**
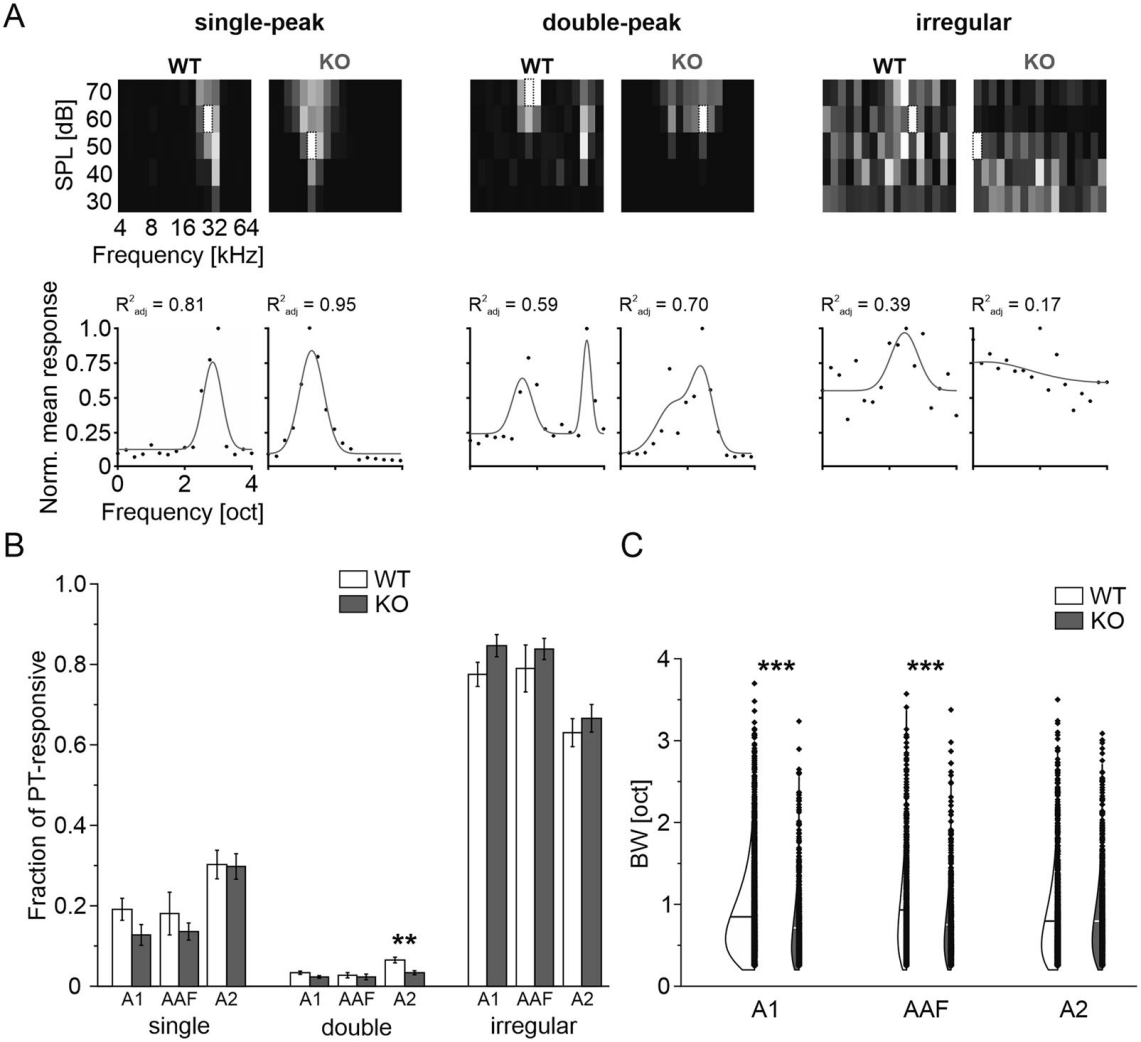
Characterization of PT response patterns. ***A***, Top: Example FRAs for each tuning type in *FMR1* KO and littermate control. Greyscale depicts normalized activity level. Dotted rectangle indicates BF. Bottom: Averaged activity across SPLs for each frequency (dots). Unimodal or bimodal Gaussian fits (line) were used to define tuning types according to their *R*^2^_adj_ value. ***B***, Fraction of PT-responsive neurons displaying single-peak, double-peak, or irregular tuning properties. Samples are FOVs with at least 100 PT-responsive neurons per subfield. ***C***, Tuning BW of single-peak neurons by subfield. S.L.W. performed experiments and analyzed data. See Extended Data Figure 1-1 for examples of original traces and deconvolution, Extended Data Figure 1-2 for widefield imaging to determine subfield borders, Extended Data Figure 1-3 for BW analysis after trace rescaling, and Extended Data Table 1-1 for statistics for panel ***B***.

To study topographic order and ensemble activity patterns of neurons within the AC of *FMR1* KO mice and WT littermates, we used two-photon imaging at single-cell level of GCaMP7f-expressing L2/3 neurons. We presented randomized PTs at increasing SPLs (4–64 kHz, 30–70 dB SPL), to characterize frequency tuning and topography. From a total of 82,141 identified WT neurons and 63,409 KO neurons, 20,757 and 11,916 neurons were responsive to PTs (see Materials and Methods for details). These were further classified as “single-peak”, “double-peak”, and “irregular”-tuned neurons (Fig. 1*A*, Extended Data Fig. 1-1), as done by Gaucher et al. (2020) before. In general, irregular-tuned neurons made up the biggest fraction, followed by single-peak neurons and double-peak neurons in both genotypes (WT: irregular, 74%; single-peak, 23%; double-peak, 4%; KO: irregular, 80%; single-peak, 17%; double-peak, 3%). Their location was aligned with the borders of AC subfields as determined by conventional widefield fluorescence imaging at low magnification. Subfield parcellation resulted in three subfields, namely, A1, AAF, and A2. In some cases, following the nomenclature from Romero et al. (2019), five subfields could be identified. To streamline analysis, in such cases, the suprarhinal auditory field was termed A2, and the dorsoposterior field and ventral posterior auditory field, if present, were added to A1. Furthermore, tone-responsive regions adjacent to subfields were added to them (Extended Data Fig. 1-2).

10.1523/ENEURO.0396-23.2024.f1-1 Figure 1-1 Response trace from an AC neuron in FMR1 KO. Uncut extracted fluorescent activity (ΔF/F, black trace) and corresponding deconvolution (red trace) during PT presentation at 60 dB SPL. Timings and length (250 ms) of presented PTs are illustrated by grey bars with the corresponding frequency depicted above. Each row denotes one repetition, containing each of the 17 PTs once. SLW performed experiments. Download Figure 1-1, TIF file.

**Figure 2.**
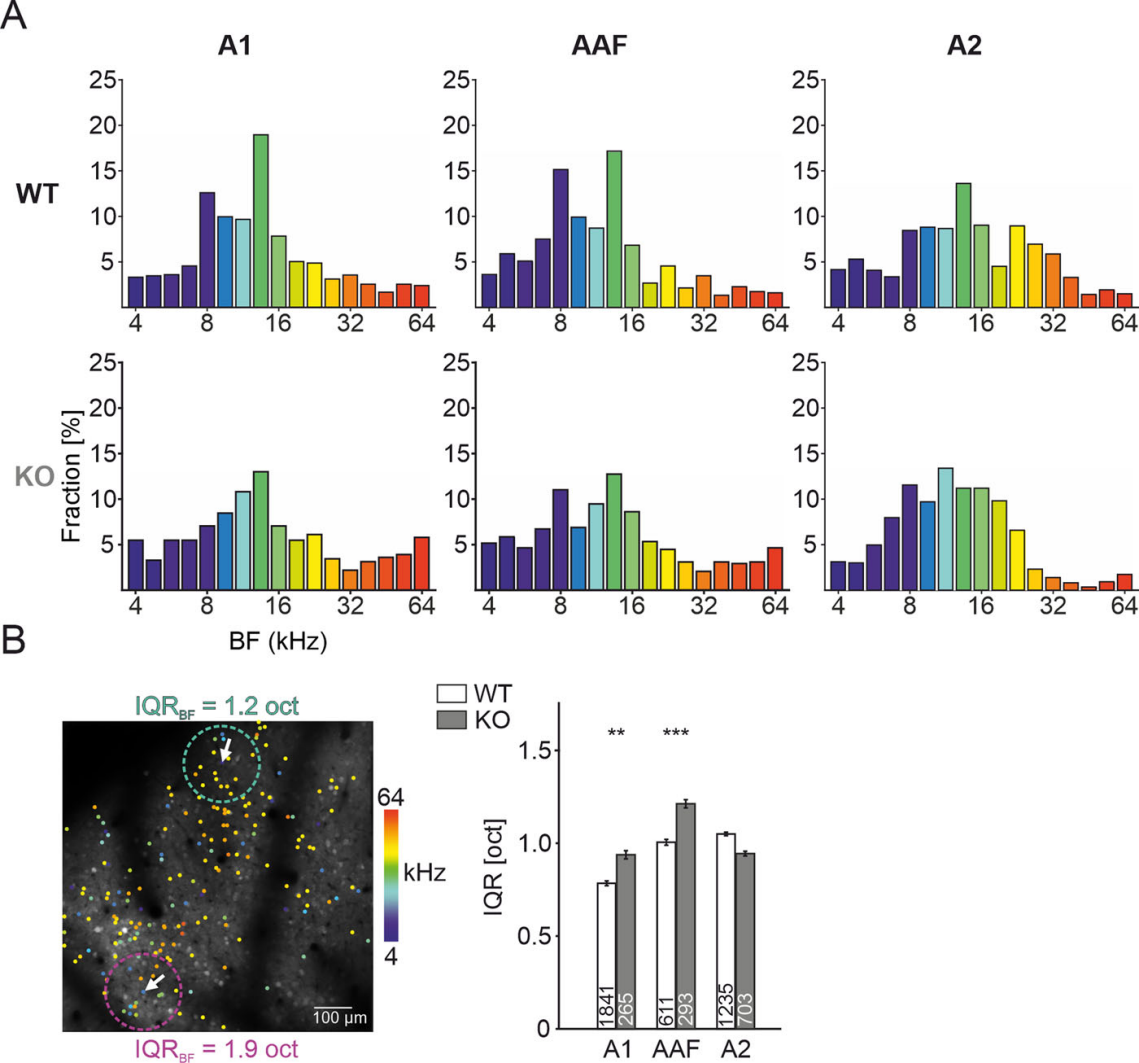
BF distributions and local BF heterogeneity. ***A***, Subfield-specific BF distribution of single-peak neurons. ***B***, Left: Two-photon maximal intensity projection of GCaMP signal across 1,000 frames in A1 of *FMR1* KO with superimposed color-coded dots denoting BFs of single-peak neurons. Local IQR calculation of BFs is shown for two example neurons (white arrows) and the respective area considered for IQR analysis (cyan and purple dotted circles). Right: Subfield-specific IQR analysis of all single-peak neurons. S.L.W. performed experiments. S.L.W. and J.J.H. analyzed data.

10.1523/ENEURO.0396-23.2024.f1-2 Figure 1-2 WF maps and subfield parcellation in FMR1 KO and littermate control. (A) Left: Cranial window with superimposed BF false-color map of a WT control. Middle: Same BF maps as left with borders of five subfields and nomenclature as in Romero et al. (2019). Right: Same map as middle and left but with borders drawn by merging DP and VPAF to A1 and adding tone responsive regions to the nearest subfield. SRAF was renamed as A2. (B) Same as (A) but for FMR1 KO. SLW performed experiments and analyzed data. Download Figure 1-2, TIF file.

**Figure 3.**
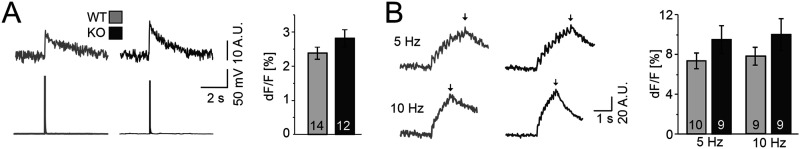
Single-cell imaging of AP-evoked Ca^2+^ amplitudes. ***A***, Left: Electrical recording (bottom) and Ca^2+^ fluorescence signals (top) of a single OGB-1-filled AC neuron in WT and KO. Right: Statistics for single AP-evoked Ca^2+^ amplitudes. Numbers in bars depict number of recorded neurons. ***B***, Left: Ca^2+^ fluorescence signals during bursts of 10 APs at 5 or 10 Hz. Right: Statistics for single AP-evoked Ca^2+^ amplitudes. Numbers in bars depict number of recorded neurons. T.C.R. performed experiments. T.C.R., S.L.W., and J.J.H. analyzed data.

10.1523/ENEURO.0396-23.2024.f1-3 Figure 1-3 BW analysis after rescaling of traces in KO animals. (A) Tuning BW of single-peak neurons by subfield. (B) Same as (A), but after rescaling of traces obtained from KO animals. SLW performed experiments and analyzed data. Download Figure 1-3, TIF file.

10.1523/ENEURO.0396-23.2024.t1-1 Table 1-1 Statistical analysis of tuning types per FOV. Only FOVs with at least 100 neurons per subfield were included in the analysis. Compared are values obtained from FMR1 KO mice and WT controls. U-test = Mann-Whitney U test. Download Table 1-1, DOCX file.

When analyzing tuning types on a FOV basis (limited to those with at least 100 PT-responsive neurons), we found no differences in the proportion between *FMR1* KO and control animals, with the exception of a minor decrease of double-peak neurons in A2 of KO mice (Fig. 1*B*, Extended Data Table 1-1). To further characterize the tuning properties of single-peak neurons, the FWHM of tuning curves was assessed. Interestingly, single-peak neurons in A1 and AAF exhibited a narrower tuning BW in *FMR1* KO compared with control (A1: WT: 0.85 oct ±0.01 oct, *n* = 2,495, KO: 0.71 oct ±0.02 oct, *n* = 637, *p *= 3.5 × 10^−13^, Mann–Whitney *U* test, AAF: WT: 0.93 oct ±0.02 oct, *n* = 744, KO: 0.75 oct ±0.02 oct, *n* = 580, *p *= 8.7 × 10^−9^, Mann–Whitney *U* test; Fig. 1*C*), whereas BW in A2 was unaltered (WT: 0.80 oct ±0.01 oct, *n* = 1,395, KO: 0.80 oct ±0.02 oct, *n* = 866, *p *= 0.06, Mann–Whitney *U* test). Neurons in A1 exhibit a BF distribution which is skewed toward low to midrange hearing frequencies (6–17 kHz; Bowen et al., 2020). This typical distribution pattern could be observed for single-peak neurons in *FMR1* KO and littermate control in A1 and AAF (Fig. 2*A*). However, in A2 of *FMR1* KO mice, midfrequencies were overrepresented (WT, 67%; KO, 57%) and high frequencies (24–64 kHz) were underrepresented (WT, 30%; KO, 14%).

Neurons in the AC exhibit a local BF heterogeneity which can span multiple octaves (Romero et al., 2019). This local BF heterogeneity is an indirect method to assess tonotopic organization (Bowen et al., 2020). We analyzed the IQR of all single-peak neurons within a 100 µm radius of a given neuron and repeated this procedure for each neuron in a subfield for single-peak neurons in *FMR1* KO and littermates (Fig. 2*B*). Single-peak neurons in *FMR1* KO exhibited an increased IQR in A1 and AAF compared with control (A1: WT: 0.8 ± 0.01 oct, KO: 0.93 ± 0.02 oct, *p *= 2.2 × 10^−3^, Mann–Whitney *U* test, AAF: WT: 0.98 ± 0.01 oct, KO: 1.22 ± 0.03 oct, *p *= 1.1 × 10^−5^, Mann–Whitney *U* test, WT vs KO, respectively; Fig. 2*B*, right), whereas in A2, IQR showed a nonsignificant tendency to be higher in control (WT: 1.06 ± 0.01 oct, KO: 0.95 ± 0.01 oct, *p *= 0.055, Mann–Whitney *U* test). These findings indicate a less structured local tonotopic organization in A1 and AAF of *FMR1* KO.

FMRP regulates several voltage-gated Ca^2+^ channel types (Fyke and Velinov, 2021). Thus, AP-evoked fluorescence changes at the soma of KO animals might differ from those observed in WT, leading to the possibility of differences in detection thresholds for the experiments described above. To test this, we prepared acute cortical slices from the brains of both genotypes and performed patch-clamp recordings of single neurons in layer 2/3 AC, filling them with OGB-1 through the patch pipette. Amplitudes of AP-induced changes in fluorescence did not differ significantly between WT and KO animals, both for single APs (WT: 0.024 ± 0.002 dF/F, *n* = 14, KO: 0.028 ± 0.003 dF/F, *n* = 12, *p* = 0.2034, unpaired *t* test; Fig. 3*A*) and trains of 10 APs evoked at 5 Hz (WT: 0.074 ± 0.008 dF/F, *n* = 10, KO: 0.095 ± 0.014 dF/F, *n* = 9, *p* = 0.1937, unpaired *t* test) or 10 Hz (WT: 0.08 ± 0.009 dF/F, *n* = 9, KO: 0.1 ± 0.016 dF/F, *n* = 9, *p* = 0.2467, unpaired *t* test; Fig. 3*B*). It should be noted though that a trend toward higher amplitudes in KO animals was observed. To assess whether this might influence our results, we employed a rescaling algorithm to decrease the Ca^2+^ trace amplitudes recorded in KO animals while keeping their noise level original (see Materials and Methods for details). We decreased the signals to 86% in accordance with fluorescence signals observed in slices when eliciting single APs. Next, we reanalyzed BW for all neurons in our dataset, as this parameter should be strongly affected by changes in activity levels. While there was a slight increase of BW in A1 (0.71 oct to 0.75 oct) and a minor reduction in A2 (0.79 oct to 0.80 oct), differences in A1 and AAF between WT and KO were still present (A1: WT: 0.85 oct ±0.01 oct, *n* = 2,495, rescaled KO: 0.75 oct ±0.01 oct, *n* = 935, *p *= 8.8 × 10^−10^, Mann–Whitney *U* test, AAF: WT: 0.93 oct ±0.02 oct, *n* = 744, rescaled KO: 0.75 oct ±0.02 oct, *n* = 589, *p *= 3.3 × 10^−9^, Mann–Whitney *U* test; Extended Data Fig. 1-3), and still no difference could be observed in A2 (WT: 0.80 oct ±0.01 oct, *n* = 1,395, rescaled KO: 0.79 oct ±0.02 oct, *n* = 956, *p *= 0.09, Mann–Whitney *U* test). We thus conclude that changes in Ca^2+^ influx into cortical AC neurons are, if present, not of concern regarding altered event detection thresholds in KO animals.

**Figure 4.**
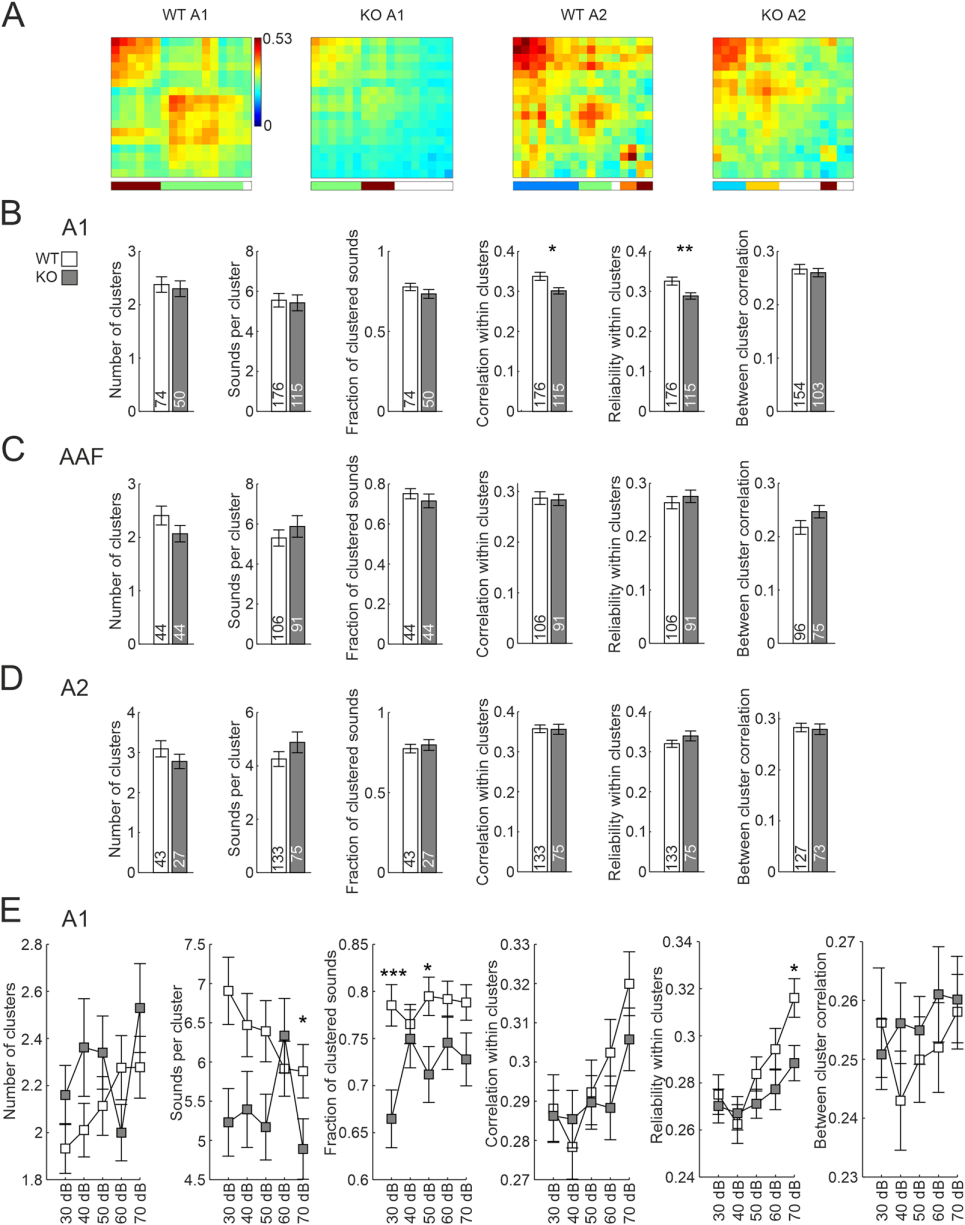
Activity clustering of neuronal ensembles in response to PTs. ***A***, Correlation matrices of 17 PT-evoked patterns after hierarchical clustering, recorded in A1 and A2 at 70 dB. Color code depicts correlation value. Vertical color bar at the bottom depicts clusters; blank stripes correspond to sounds that are not part of clusters. The diagonal depicts mean correlation across repetitions, thus showing reliability of the network. ***B***, Statistics for sound cluster analysis for 70 dB PT sounds for A1. Numbers in bars depict *n*-number, either FOVs or sound clusters. ***C***, Same as ***B***, but for data recorded in AAF. ***D***, Same as ***B***, but for data recorded in A2. ***E***, Statistics for sound cluster analysis for PTs played at different SPLs in A1. S.L.W. performed experiments. J.J.H. analyzed data. See Extended Data Tables 4-1 and 4-2 for statistics.

Next, we analyzed activity correlations and neuronal ensembles within the three AC subfields in response to different sounds. For this purpose, 17 PTs, 34 AM tones with two different modulation frequencies (same carrier frequencies as PT set), and 13 different complex sounds were presented to the animals. Sound-evoked responses were grouped into clusters based on the activity correlations of all neurons within an observed FOV, first concentrating on the set of 17 PTs (Fig. 4*A*). The number of sound clusters per FOV, the number of sounds per cluster, and the overall fraction of clustered sounds did not differ between genotypes in any of the three subfields (Fig. 4*B–D*). However, specifically in A1, the mean correlation within clusters and the reliability of neuronal activity patterns in response to repetitions of the same sound (diagonal of correlation matrices for a given cluster) were lower in KO mice. Correlation values for sounds not within the same cluster (a measure of cross talk between different neuronal ensembles) did not differ, indicating that the decrease in correlation strength within neuronal ensembles was not accompanied by a decrease in specificity (Extended Data Table 4-1). When repeating these experiments at different dB levels 1 d later (dataset also analyzed above for tuning, BW, and local heterogeneity analyzes), lower reliability in KO was confirmed for 70 dB, though lower correlation within clusters only in tendency (Fig. 4*E*, Extended Data Table 4-2). Testing other dB levels did not result in differences.

10.1523/ENEURO.0396-23.2024.t4-1 Table 4-1 Statistical analysis of AC ensemble activity in response to 17 PTs. Compared are values obtained from FMR1 KO mice and WT controls. s. = sounds, c. = clusters, corr. = correlation, rel. = reliability, T-test2 = unpaired t-test, U-test = Mann-Whitney U test. Download Table 4-1, DOCX file.

10.1523/ENEURO.0396-23.2024.t4-2 Table 4-2 Statistical analysis of AC ensemble activity in A1 in response to 17 PTs played at different SPLs. Compared are values obtained from FMR1 KO mice and WT controls. s. = sounds, c. = clusters, corr. = correlation, rel. = reliability, T-test2 = unpaired t-test, U-test = Mann-Whitney U test. Download Table 4-2, DOCX file.

**Figure 5.**
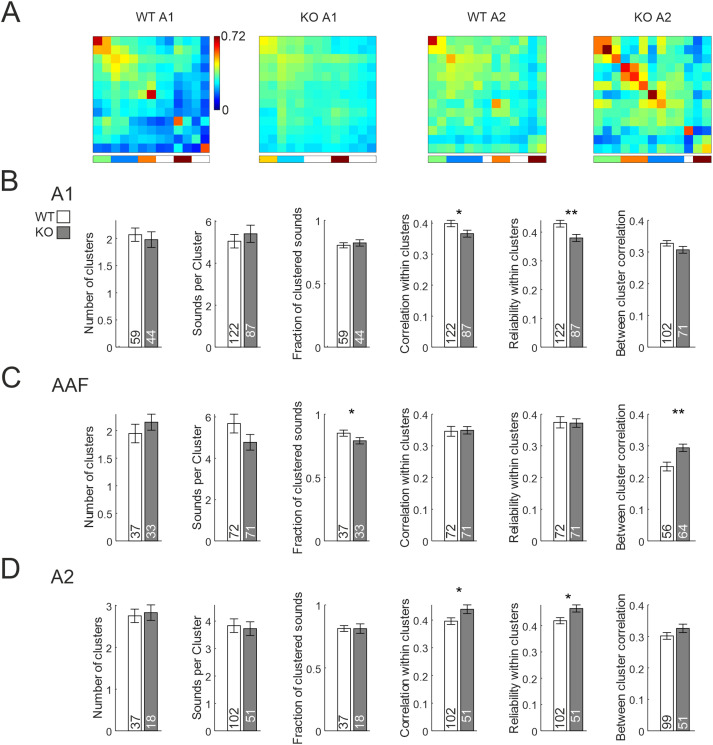
Activity clustering of neuronal ensembles in response to complex sounds. ***A***, Correlation matrices of 13 complex sound-evoked patterns after hierarchical clustering, recorded in A1 and A2. Color code depicts correlation value. Vertical color bar at the bottom depicts clusters; blank stripes correspond to sounds that are not part of clusters. The diagonal depicts mean correlation across repetitions, thus showing reliability of the network. ***B***, Statistics for sound cluster analysis for A1. Numbers in bars depict *n*-number, either FOVs or sound clusters. ***C***, Same as ***B***, but for data recorded in AAF. ***D***, Same as ***B***, but for data recorded in A2. S.L.W. performed experiments. J.J.H. analyzed data. See Extended Data Figure 5-1 for examples of correlation matrices for AAF and Extended Data Table 5-1 for statistics.

Analyzing network responses to 13 complex sounds (animal vocalizations and artificial mouse vocalization mimics) revealed similar differences as for the PT set regarding correlation and reliability for A1 (Fig. 5*A,B*, Extended Data Table 5-1), but interestingly further differences between the genotypes in AAF and A2. In AAF, the fraction of clustered sounds was slightly lower in KO mice, but more importantly, the between-cluster correlation was higher, indicating less specificity of network activity patterns in response to different sounds (Fig. 5*C*, Extended Data Fig. 5-1). In A2, correlation and reliability were, in contrast to A1, increased in KO mice (Fig. 5*D*).

10.1523/ENEURO.0396-23.2024.f5-1 Figure 5-1 Correlation matrices of 13 complex sound-evoked patterns after hierarchical clustering, recorded in AAF. Color code depicts correlation value. Vertical color bar at the bottom depicts clusters, blank stripes correspond to sounds that are not part of clusters. The diagonal depicts mean correlation across repetitions, thus showing reliability of the network. Download Figure 5-1, TIF file.

10.1523/ENEURO.0396-23.2024.t5-1 Table 5-1 Statistical analysis of AC ensemble activity in response to 13 complex sounds. Compared are values obtained from FMR1 KO mice and WT controls. s. = sounds, c. = clusters, corr. = correlation, rel. = reliability, T-test2 = unpaired t-test, U-test = Mann-Whitney U test. Download Table 5-1, DOCX file.

**Figure 6.**
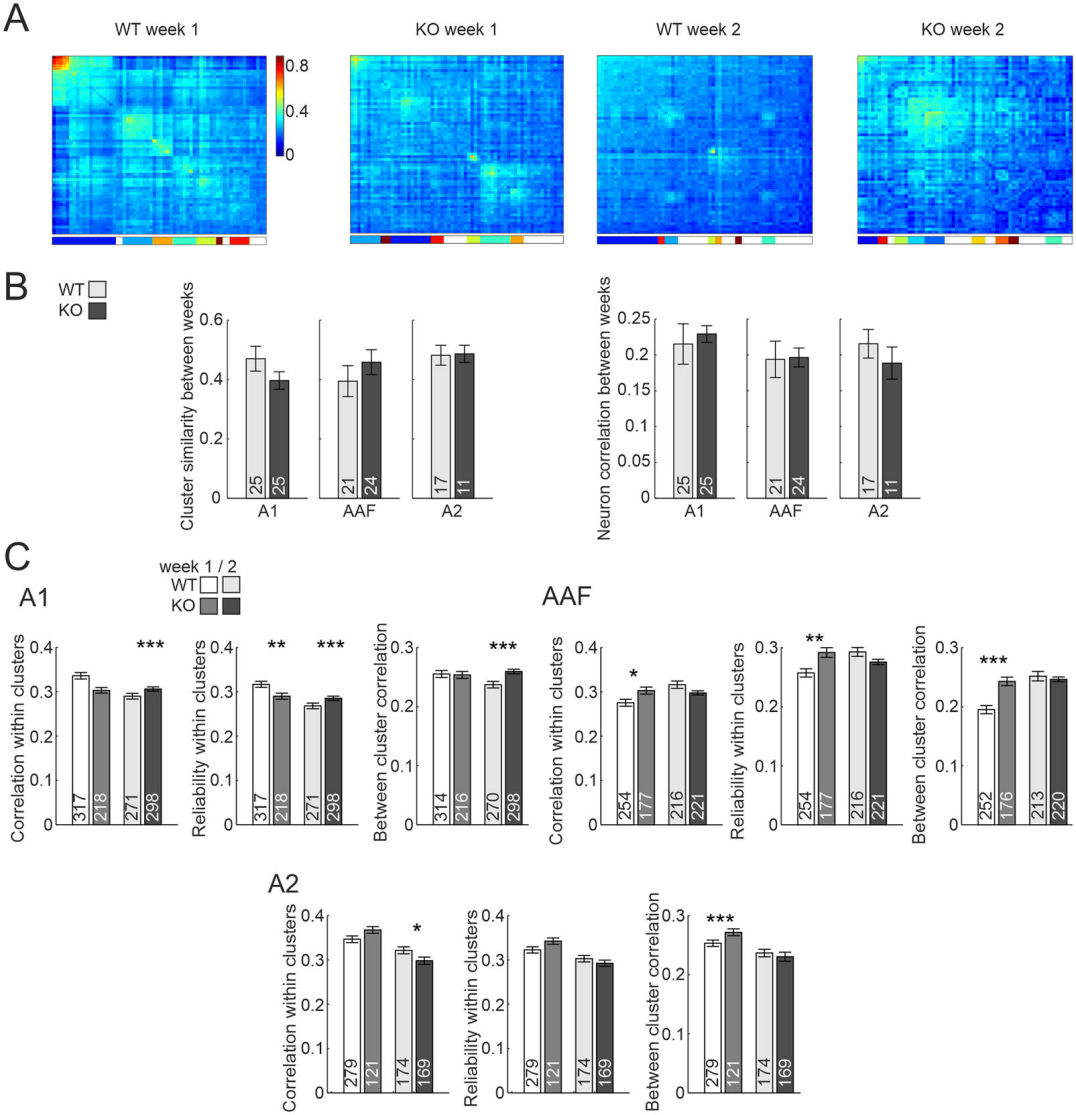
Activity clustering of neuronal ensembles in response to simple and complex sounds. ***A***, Correlation matrix of 64 sound-evoked patterns (17 patterns for each of PTs, AM tones with 20 Hz modulation, AM tones with 40 Hz modulation, and 13 patterns for complex sounds) after hierarchical clustering, recorded in A1. Color code depicts correlation value. Vertical color bar at the bottom depicts clusters; blank stripes correspond to sounds that are not part of clusters. The diagonal depicts mean correlation across repetitions, thus showing reliability of the network. ***B***, Cluster similarity (left) and neuron correlation (right) between 2 d separated by 1 week. ***C***, Correlation values within clusters, reliability within clusters, and correlation values between clusters. S.L.W. performed experiments. J.J.H. analyzed data. See Extended Data Figure 6-1 for images of recorded neurons 1 week apart, Extended Data Figure 6-2 for statistics between the two recording time points and Extended Data Table 6-1–6-4 for statistics.

To assess the stability of network activity features, experiments were repeated after 1 week (see Materials and Methods for details; Fig. 6, Extended Data Fig. 6-1). Correlation analysis was performed across the complete set of 17 PTs, 34 AM tones, and 13 complex sounds. The between-week similarity of the content of sound clusters for a given FOV did not differ between the genotypes across all subfields (A1: WT: 0.47 ± 0.04, *n* = 25, KO: 0.40 ± 0.03, *n* = 25, *p *= 0.1578, unpaired *t* test, AAF: WT: 0.40 ± 0.05, *n* = 21, KO: 0.46 ± 0.04, *n* = 24, *p *= 0.3405, unpaired *t* test, A2: WT: 0.48 ± 0.03, *n* = 17, KO: 0.49 ± 0.03, *n* = 11, *p *= 0.9204, unpaired *t* test; Fig. 6*B* left), implying a similar stability of sound categorization for the two genotypes. Furthermore, when limiting the dataset to neurons observed on both experimental days, the correlation between the two was similar for WT and KO as well (A1: WT: 0.22 ± 0.03, *n* = 25, KO: 0.23 ± 0.01, *n* = 25, *p *= 0.6497, unpaired *t* test, AAF: WT: 0.19 ± 0.03, *n* = 21, KO: 0.20 ± 0.01, *n* = 24, *p *= 0.9237, unpaired *t* test, A2: WT: 0.22 ± 0.02, *n* = 17, KO: 0.19 ± 0.02, *n* = 11, *p *= 0.3884, unpaired *t* test; Fig. 6*B* right). Thus, the basic stability of sound feature analysis appears to be largely unaffected in *FMR1* KO mice within the time period observed. It should however be noted that again differences were apparent regarding correlation and reliability within and between clusters (Fig. 6*C*). For A1, all values were higher for KO animals in the second, but not the first week of experiments. In contrast, in AAF, higher values were observed only in the first week. For A2, correlation within clusters was lower in the second week in KO animals, but correlation between clusters was higher in the first week, overall showing a tendency of lower correlation values for the second week for KO animals (Extended Data Table 6-1, Extended Data Table 6-2). Statistical analysis across the FOVs observed in both weeks revealed that these differences were the results of changes in almost exclusively either WT or KO, depending on the subfields imaged. This was expressed in a decrease in correlations in A1 for WT, an increase for WT in AAF, and a decrease in A2 for KO (Extended Data Fig. 6-2, Extended Data Table 6-3, Extended Data Table 6-4). In summary, while the general stability of sound categorization and network composition appeared to be unaffected in *FMR1* KO mice, either activity correlations and response reliability within and between neuronal ensembles underwent alterations within 1 week of observation that did not occur in WT or alterations observed in WT were not present in KO, depending on the subfields.

10.1523/ENEURO.0396-23.2024.t6-1 Table 6-1 Statistical analysis of AC ensemble activity in response to 17 PTs, 34 AM-modulated tones and 13 complex sounds, with data collected in the first week of experiments. Compared are values obtained from FMR1 KO mice and WT controls. s. = sounds, c. = clusters, corr. = correlation, rel. = reliability, T-test2 = unpaired t-test, U-test = Mann-Whitney U test. Download Table 6-1, DOCX file.

10.1523/ENEURO.0396-23.2024.t6-2 Table 6-2 Statistical analysis of AC ensemble activity in response to 17 PTs, 34 AM-modulated tones and 13 complex sounds, with data collected in the second week of experiments. Compared are values obtained from FMR1 KO mice and WT controls. s. = sounds, c. = clusters, corr. = correlation, rel. = reliability, T-test2 = unpaired t-test, U-test = Mann-Whitney U test. Download Table 6-2, DOCX file.

10.1523/ENEURO.0396-23.2024.t6-3 Table 6-3 Statistical analysis of AC ensemble activity in response to 17 PTs, 34 AM-modulated tones and 13 complex sounds, comparing values obtained from WT mice across one week. s. = sounds, c. = clusters, corr. = correlation, rel. = reliability, T-test = paired t-test, T-test2 = unpaired t-test, U-test = Mann-Whitney U test. Download Table 6-3, DOCX file.

10.1523/ENEURO.0396-23.2024.t6-4 Table 6-4 Statistical analysis of AC ensemble activity in response to 17 PTs, 34 AM-modulated tones and 13 complex sounds, comparing values obtained from FMR1 KO mice across one week. s. = sounds, c. = clusters, corr. = correlation, rel. = reliability, T-test = paired t-test, T-test2 = unpaired t-test, U-test = Mann-Whitney U test. Download Table 6-4, DOCX file.

10.1523/ENEURO.0396-23.2024.f6-1 Figure 6-1 Neurons can be identified across experimental days. (A) Example section of a FOV in FMR1 KO in week 1 and the same section in week 2. Scale bar: 50 µm. (B) Magnified sections from the rectangles in (A) in week 1 and 2, respectively. Scale bar: 10 µm. (C) Same as (A), but for an example obtained from a WT animal. (D) Same as (B) but showing the magnifications from (C). Download Figure 6-1, TIF file.

10.1523/ENEURO.0396-23.2024.f6-2 Figure 6-2 Alteration in correlation values between sound-evoked activity patterns across one week. 64 sound-evoked patterns (17 patterns for each of PTs, AM tones with 20Hz modulation, AM tones with 40 Hz modulation, and 13 patterns for complex sounds) were analyzed. Numbers in bars depict n-number (sound clusters). SLW performed experiments. JJH analyzed data. Download Figure 6-2, TIF file.

In summary, frequency-specific alterations of network ensemble activity in KO mice were observed only in A1, while alterations in the processing of complex sounds were present in AAF and A2 as well, though expressed in a subfield-specific manner. The basic stability of sound feature analysis across 1 week was unaffected, but correlation values within sound clusters appeared to change within this time in both genotypes, depending on the subfield observed.

## Discussion

In this study, we performed two-photon imaging of L2/3 neurons in AC of *FMR1* KO mice and WT littermate controls to analyze frequency tuning and topography as well as ensemble activity in a mouse model of FXS. We found a reduction in tuning BW and a decreased topographic order of frequency representation in most AC subfields, as well as alterations in ensemble activity, foremost manifested in decreased correlations in A1, increased correlations in A2, and decreased sound specificity in AAF.

Overall, 25% of active neurons were PT-responsive in WT animals, which is in a similar range to a previous report (25–35%; Bowen et al., 2020) but is less compared with Gaucher et al. (2020), who reported 44% of WT neurons being PT-responsive. There are several technical factors that could account for this discrepancy. Gaucher et al. (2020) used GCaMP6m as Ca^2+^ sensor, which has a lower sensitivity and smaller peak fluorescence compared with jGCaMP7f used in this study (Chen et al., 2013; Dana et al., 2019). The resulting higher signal-to-noise ratio might have led to more neurons being identified. Typically, neurons with low signal-to-noise ratio exhibit weak tuning (Gaucher et al., 2020; Extended Data Fig. 5-1), shifting the distribution imaged with GCaMP7f toward non-PT-responsive neurons. Furthermore, we used suite2p version 0.10.1 with improved cell detection which was not used by Gaucher et al. (2020), as it was not released at publication date, again increasing the number of cells with low signal-to-noise ratios. These aspects might, together with differences in classification methods used, also account for a lower amount of single-peak neurons in our dataset (23%) compared with Gaucher et al. (2020), where 60% of PT-responsive neurons display a single-peak tuning pattern. As high signal-to-noise-ratio neurons typically exhibit the strongest responses (Issa et al., 2014; Bowen et al., 2020; Gaucher et al., 2020), the preference of them being detected over neurons with weaker responses shifts their dataset toward highly tuned neurons compared with ours.

Interestingly, we found that neurons classified as single-peak actually displayed a reduction in tuning width, which is in contrast to the findings by Rotschafer and Razak (2013), who reported less precise tuning. Several factors might explain this discrepancy. First, Rotschafer and Razak (2013) pooled data across all layers with only 24 and 12 units (20 and 12% of all recordings) in L2/3 for WT and *FMR1* KO, respectively, which hampers any conclusions regarding L2/3. Second, they conducted experiments with anesthetized mice, which influences the tuning properties of AC neurons, as somatostatin interneuron activity is vastly reduced during anesthesia (Adesnik et al., 2012), which in turn affects PV cell activity and consequently tuning BW. Whereas deletion of *FMR1* in somatostatin interneurons did not result in an ASD-like behavioral phenotype, *FMR1* deletion in PV interneurons caused typical behavioral abnormalities (Kalinowska et al., 2022). Furthermore, PV cells are decoupled from pyramidal neurons in early development in the somatosensory cortex in *FMR1* KO mice (Kourdougli et al., 2023). Therefore, the observed narrowing of tuning BW in *FMR1* KO in this study is possibly caused by an altered PV cell activity, enhancing lateral inhibition despite a reduced number of PV interneurons found in *FMR1* KO and FXS patients (Wen et al., 2018; Kourdougli et al., 2023).

One of the major advantages of using two-photon imaging to study neuronal activity is the ability to precisely analyze the topographical order of single neurons within a network. While earlier works in the AC reported a quite unstructured single-cell tonotopy, displaying order only at a large scale (Bandyopadhyay et al., 2010; Rothschild et al., 2010; reviewed in Kanold et al., 2014), recent studies have favored a moderate tonotopy (Romero et al., 2019; Gaucher et al., 2020). Our data revealed a higher local tuning heterogeneity in A1 and AAF of *FMR1* KO for single-peak neurons, pointing toward a worse tonotopic organization in these subfields. This is in line with a reduction in tonotopic organization in the primary AC fields observed in a valproic acid-induced rat model of ASD (Anomal et al., 2015), altogether implying that a less precise frequency-related AC topography might be a common occurrence in different forms of ASD-associated disorders. The most likely explanation for this not being observed for A2 in *FMR1* KO is a narrower BF distribution in this subfield, which is a factor toward lower heterogeneity. While the direct cause of altered topographic organization and FRA shapes in *FMR1* KO remain unclear at this point, they are probably caused by altered critical period plasticity during development, as observed in the AC (Kim et al., 2013) as well as the barrel cortex (Harlow et al., 2010). Alterations in topography might also be caused by a change in local connectivity patterns and integration from a broader frequency range. In V1, neurons with similar orientation tuning exhibit higher synaptic connection probabilities and stronger connections, which might be similar in the AC (Ko et al., 2011; Cossell et al., 2015; Gaucher et al., 2020).

Analyzing ensemble activity revealed PT-related changes in *FMR1* KO mice as well. In A1, reliability of network responses as well as correlations between ensembles within a given sound cluster was lower. This was not observed in other subfields, which is in line with A1 coding particularly strong for the spectral component of sounds (Sołyga and Barkat, 2021, 2022), but also makes it difficult to relate these results to changes in BW and topographical order reported above, which also affected AAF. In contrast, ensemble activity in response to complex sounds was altered in AAF and A2 as well. In AAF, which is associated with processing of temporal features of sounds (Sołyga and Barkat, 2019, 2021, 2022), the specificity of ensemble activity between sound clusters was impaired. For A2, which is generally associated with encoding complex sounds, such as vocalizations (Carruthers et al., 2015) and harmonic sounds (Kline et al., 2021), correlations and reliability were actually higher in *FMR1* KO mice. This is also interesting because task-relevant sounds modulate activity levels and elicit categorization stronger in A2 than in A1 (Atiani et al., 2014; Yin et al., 2020), which might be related to difficulties in decision-making reported in ASD (Luke et al., 2011; Geurts et al., 2020). These alterations in AC activity patterns might contribute to problems in speech perception and auditory hypersensitivity observed in FXS (Fidler et al., 2007; Finestack et al., 2009; Ethridge et al., 2017; McCullagh et al., 2020) and ASD (Alcántara et al., 2004; Groen et al., 2009; DePape et al., 2012; Schelinski and von Kriegstein, 2020). In line with this, *FMR1* KO rats display degraded responses to speech sounds in A1, AAF, and A2 (Engineer et al., 2014). Interestingly, ASD individuals display abnormal auditory temporal and speech processing, but intact spectral processing (Groen et al., 2009), which might lead to the conclusion that slight alterations in activity correlation found in our study are less consequential for auditory processing compared with the decreased sound specificity found in AAF. This view is also supported by another study in *FMR1* KO rats, which reported diminished temporal but enhanced spectral integration of sound intensity (Auerbach et al., 2021). However, Schelinski et al. (2017) reported changes in specifically vocal pitch processing in ASD, but not for other forms of pitch. Thus, it might be difficult to directly relate alterations in PT processing in *FMR1* KO mice to sound frequency processing in the context of speech.

Cortical circuits are known to undergo representational drift, with neurons changing their response properties to stimuli while keeping overall representation of the stimuli constant to a large degree (Pérez-Ortega et al., 2021; Aschauer et al., 2022; Chambers et al., 2022). ASD is often associated with preservative thinking and repetitive behavior (American Psychiatric Association, 2013), and in children with ASD, learning is associated with more stable rather than plastic neural representation (Liu et al., 2023). Thus, investigating representational drift in *FMR1* KO mice is a promising endeavor to uncover the underlying causes on a circuitry level. In fact, developmental plasticity is grossly impaired, as demonstrated by unaltered PT representation in early and late exposed *FMR1* KO mice (Kim et al., 2013), and reduced adaptation to stimuli has been observed in the AC (Lovelace et al., 2016) as well as the somatosensory cortex (He et al., 2017). However, both the correlation of neuronal activity of the same set of neurons observed across 1 week in response to various sounds and the similarity of sound clusters between the 2 experimental days did not differ from WT controls, arguing against changes in either representational drift or general cortical sound representation. Nevertheless, changes in plasticity were observed when analyzing sound clusters in more detail. In A1 and AAF, correlations and reliability between sounds and repetitions either decreased or increased during our observation period in WT while staying constant in KO mice. In contrast, these values decreased in A2 in KO mice, but not in WT. These observations should only be regarded as a first assessment of network stability in *FMR1* KO AC and need to be investigated in more detail, employing, for example, repeated sound stimulation over an extended time period. This is also important in the context of learning defects reported in the tactile (Arnett et al., 2014), visual (Goel et al., 2018; Kissinger et al., 2020), spatial (Nolan and Lugo, 2018), or auditory domain (Kim et al., 2013; S. Yang et al., 2014) in *FMR1* KO. In future experiments, combining large-scale network activity imaging with learning paradigms will be a crucial step in relating the alterations in a passive listening situation reported here with their behavioral consequences. This approach would also help to investigate whether the changes observed in PT processing are related to the less precise topography in *FMR1* KO. Such a correlation has been described in the visual cortex in *FMR1* KO, relating orientation-tuning deficits and reduced activity of PV interneurons to delayed learning of a visual discrimination task (Goel et al., 2018).

A wide array of network alterations which might underlie the ensemble activity changes observed here have been reported in cortical circuits of *FMR1* KO rodents. Studies in the somatosensory cortex revealed deficits in local excitatory drive targeting fast-spiking inhibitory neurons (Gibson et al., 2008), increased excitation–inhibition ratio (Antoine et al., 2019), weak Layer 4 to Layer 3 connectivity (Bureau et al., 2008), broadened receptive fields in L2/3 (Juczewski et al., 2016), absent pruning of connectivity in Layer 5 (Patel et al., 2014), abnormal synaptogenesis (Booker et al., 2019) and spine development (Nimchinsky et al., 2001), weak callosal projections (Zhang et al., 2021), and increased neuronal noise (Bhaskaran et al., 2023). Furthermore, interneurons and pyramidal neurons as well as pyramidal neurons themselves are desynchronized (Paluszkiewicz et al., 2011; Kourdougli et al., 2023), and sensory encoding precision is reduced (Domanski et al., 2019). In the visual cortex, hyperconnectivity and increased dendritic complexity (Haberl et al., 2015; C. Yang et al., 2022) as well as increased spine turnover (Ishii et al., 2018) were reported. Similar alterations have been observed in AC circuits as well, including an increase in spine density, with immature spine morphology (Lee et al., 2019), altered GABAergic signaling (Song et al., 2022), abnormal perineuronal net development around PV interneurons (Lovelace et al., 2016; Wen et al., 2018), and local hyperconnectivity, but long-range hypoconnectivity (Haberl et al., 2015). Interestingly, while forebrain-specific KO of *FMR1* leads to similar changes in PV cells and perineuronal net structure, gamma band oscillations, typically associated with synchronized activity upon auditory stimulation, were not altered in contrast to decreased synchronization in the global KO (Lovelace et al., 2019). This points toward an important role of brainstem nuclei in *FMR1* KO-related deficits and ASD (Seif et al., 2021; Jure, 2022), which might be the cause of some of the observations reported here, as abnormal activity patterns are transmitted via the thalamus up to the AC. We previously reported altered topography and sound clustering in mice missing the α_2_δ3 calcium channel subunit (Wadle et al., 2022), an ASD-associated mouse model that displays temporal sound-processing defects and synaptic transmission abnormalities in brainstem nuclei (Pirone et al., 2014; Bracic et al., 2022). However, cortical changes observed in this study differed regarding their subfield specificity, displaying decreased correlations in A1 in response to PTs and complex sounds, as well as a reduced heterogeneity of frequency tuning in AAF and A2. These differences highlight the wide array of neuronal alterations associated with ASD, but also point toward common phenotypes concerning auditory processing. Further studies investigating AC activity in multiple ASD mouse models are needed to pinpoint these further. Furthermore, the involvement of neuronal circuits outside of the auditory system needs to be considered as well, as highlighted by a recent study showing a developmental delay in temporal processing in *FMR1* KO mice to be located in the frontal cortex rather than in the AC (Croom et al., 2023).

In conclusion, we demonstrate altered sound-evoked activity in AC networks of *FMR1* KO mice, expressed in decreased tonotopy and changes in correlations within and between neuronal ensembles, depending on the subfield analyzed. These results provide detailed insight into AC activity in a mouse model of FXS and thus help to understand the causes of sound-processing defects in ASD-associated disorders.

## Synthesis

Reviewing Editor: Christine Portfors, Washington State University Decisions are customarily a result of the Reviewing Editor and the peer reviewers coming together and discussing their recommendations until a consensus is reached. When revisions are invited, a fact-based synthesis statement explaining their decision and outlining what is needed to prepare a revision will be listed below. The following reviewer(s) agreed to reveal their identity: Khaleel Razak.

Thank you for addressing all the prior comments. The manuscript is considerably improved with the new analysis and removal of discussions regarding critical period plasticity. One very minor comment is that in ASD, 'abnormal' sensitivity and not just 'increased' sensitivity to sounds is present (eg., intro line32).

## Author Response

Rebuttal letter for manuscript "Topography and ensemble activity in auditory cortex of a mouse model of Fragile-X-Syndrome" Synthesis Statement for Author (Required): The reviewers agree that this is a potentially interesting study. One of the main conclusions of the paper is there is abnormal plasticity in the Fmr1 KO auditory cortex over the course of 2 weeks. However, it is unclear what is driving the plasticity as there are no controlled experiential factors over the weeks of recordings. This is a major concern. The reviewers also agree that the analysis of the frequency tuning is not rigorousl and this substantially limits the interpretation. The reviewers agree that substantial new analyses are warranted and additional experiments are necessary. Otherwise, the claims in the paper need to be substantially reduced. We would like to thank the reviewers and editors for their constructive input to our manuscript. We fully agree that our conclusions regarding plasticity were not warranted. We have thus removed most aspects regarding this analysis from the manuscript and concentrate mostly on data obtained in the first week of experiments. These analyses have been refined, leading to more interesting findings than in our first version of the work. Further details can be found below. Regarding the analysis of frequency tuning, we have revised some aspects considerably, yet also think that our analysis of bandwidth is scientifically sound. These points are discussed below. We hope that these revisions make our manuscript suitable for publication in eNeuro. Additional comments: FXS is a specific genetic cause of intellectual disorder and autism-associated behaviors. The Fmr1 KO mice is a model for FXS. Therefore broader conclusions about how this study provides information about ASD auditory processing should be reduced across the full manuscript. The relation of our results to ASD has been significantly toned down. The percentage of single peaked frequency tuned neurons in primary auditory cortex and anterior auditory field seems very low, compared to single unit recordings and even to other calcium imaging studies. While an explanation is provided about GCaMP6 vs. GCaMP7, the description of these technical differences in the discussion is confusing. The text suggests that GCaMP6 has lower sensitivity, but higher SNR, and the current study picked up less tone responsive neurons due to lower SNR. In a number of analyses, data across subfields are combined, and in others they are shown separately. In a number of places, anecdotal phrases such as 'small increase' or 'skewed toward' or 'maybe not as prominent' or 'tendency lower' are used. These have to be replaced with tighter statistical tests and information. For bandwidth measurements, it is more appropriate to use Q values (bw normalized to best or characteristic frequencies) as any difference in tuning distributions can lead to apparent bandwidth distributions across genotypes. The concluding paragraph in discussion refers to 'reduced number of frequency tuned neurons', but it is unclear where the statistics were shown for that. How subfield parcelation was achieved when the vast majority of neurons were broadly/irregularly tuned is unclear. We understand the confusion regarding our explanation of the low number of single peaked neurons. What we meant to express is that in a situation where - relatively speaking - only neurons with strong signals are identified (such as when using GCaMP6), these neurons will more likely show single peak tunings, as single peaked neurons display strong responses (Issa et al., 2014; Bowen et al., 2020; Gaucher et al., 2020). Increasing our detection threshold using GCaMP7 thus might have led us to detect more neurons overall, but also those with less well-defined tuning. We have made this clearer in the discussion now. We appreciate the comments regarding the anecdotal statements and missing statistical tests throughout our manuscript. We have removed most of these statements and performed statistical analysis on the fraction of neurons with different tuning types. We now conclude that there are no/very few differences in tuning types observed between the genotypes. To declutter our work, we also have removed all instances of data pooled across the three subfields. We acknowledge the suggestion to use Q-values as a factor of bandwidth measure and agree that any difference in tuning distribution can lead to shifts in bandwidth if measured in absolute frequency. However, since we used octave bandwidth (N) and Q-values are directly inverse proportional by the formula below, we believe to have covered this issue already. Q = √2N 2N−1 Nevertheless, we calculated Q-values for all single-peak neurons (A1: WT: 2.32 {plus minus} 0.03, KO: 2.80 oct {plus minus} 0.06 oct, p = 3.4 x 10-13, Mann-Whitney U-test, AAF: WT: 2.18 oct {plus minus} 0.05, KO: 2.59 oct {plus minus} 0.06 oct, p = 8.5 x 10-9 , Mann-Whitney U-test, A2: WT: 2.59 {plus minus} 0.04, KO: 2.43 {plus minus} 0.04, p = 0.06, Mann-Whitney U-test) and could not detect any differences to our analysis with octave bandwidth (Rebuttal figure 1). As octave bandwidth is a common way of analysis (Romero et al., 2019), we would like to keep it as our method of choice in our manuscript. Nevertheless, we are open to the discussion whether Q-values should be added in addition, though we think it does not add further information. Rebuttal figure 1: Bandwidth in octaves (A) and Q-values (B) of single peaked neurons in AC. Regarding subfield parcellation, it is well-documented that a global AC tonotopy is present even in experimental environments where both local tuning heterogeneity of single neurons and the amount of untuned neurons are high (Bandyopadhyay et al., 2010; Rothschild et al., 2010). The global tonotopy picked up by our widefield imaging is thus a product of slight differences in frequency preference of different parts of the AC subfields. Neurons with no frequency-related tuning (or tunings that deviate from the predominantly present tuning in a given area) would contribute noise to our recordings, leading to a somewhat fuzzy, but still identifiable subfield-related tonotopy. The suggestion in the conclusion that there is more experience dependent change in KO mice is also not strongly supported by data (stats for decrease in A2, for e.g.). But more importantly, it is unclear what 'experience' means here as there was no controlled manipulation between 1st and 2nd recording. The discussion talks about critical period plasticity in relation to this, but the present finding seems unrelated to the developmental plasticity papers that typically provide a specific tone experience during development, and observe changed representation for that tone. The experience dependency of recordings done a week apart needs some controlled task or experience manipulation to be convincing. Related to this, the Result narrative on the cluster analysis was difficult to follow with 'genotype x multiple regions x weeks x sound types' interactions seemingly producing rather variable outcomes that fail to suggest any specific pathophysiology in Fmr1 KO mice. As recordings were done only twice (1 week apart), whether these changes are stable or not is not known. We very much agree that our analysis and conclusions of this part of our work have focused on the wrong aspects. Experience-dependent plasticity needs to be investigated in a more controlled manner. We have fundamentally revised the ensemble activity-related parts of the manuscript. They now focus on differences between the genotypes that are observed the first time we present the acoustic stimuli to the animals. The analysis on complex sounds now also includes 2 mouse vocalization mimics and a mix of natural animal vocalizations to increase the number of different sounds analyzed in this group. This has led to new results which are included in the revised version of the manuscript. In summary, in the first week of experiments we observe lower correlations of neurons in A1 in FMR1 KO mice, higher correlations in A2, and lower sound specificity in AAF. We have included a more thorough discussion of these findings compared to the first version of the manuscript. This process also greatly reduced the number of comparisons between different groups, which streamlines our manuscript significantly. We are keeping the comparison between the two time points of imaging only for one analysis at the end of the Results section and phrase it largely as an outlook for future experiments. We think that the observation that changes across this time period are happening in a subfield- and genotype-specific manner is still interesting, and should be included in our manuscript, even though we are not basing any strong conclusions on these findings. Statistics in Figure 3 - underpowered? There seems to be a clear trend towards a difference in signal which could impact how we view the genotype differences. How were the sample sizes estimated to be used? The concerns raised by the reviewer are valid. However, it is difficult to estimate a needed sample size, given that a high number of parameters (expression of GCaMP, Ca2+ buffering in different neuron types, depth within the cortex, ...) influence the size of calcium signals. We thus used a different strategy to assess whether the potential difference in Ca2+ signals might have influenced our results. We employed a rescaling algorithm to decrease the Ca2+ trace amplitudes recorded in KO animals while keeping their noise level original. We decreased the signals to 86% in accordance with fluorescence signals observed in slices when eliciting single APs. Next, we re-analyzed bandwidth for all neurons in our dataset, as this parameter should be strongly affected by changes in activity levels. While there was a slight increase of bandwidth in A1 (KO → rescaled KO: 0.71 oct → 0.75 oct) and a minor reduction in A2 (KO → rescaled KO: 0.79 oct → 0.80 oct), differences in A1 and AAF between WT and KO were still present (A1: WT: 0.85 oct {plus minus} 0.01 oct, rescaled KO: 0.75 oct {plus minus} 0.01 oct, p = 8.8 x 10-10 , Mann-Whitney U-test, AAF: WT: 0.93 oct {plus minus} 0.02 oct, rescaled KO: 0.75 oct {plus minus} 0.02 oct, p = 3.3 x 10-9 , Mann-Whitney U-test) and still no difference could be observed in A2 (WT: 0.80 oct {plus minus} 0.01 oct, rescaled KO: 0.79 oct {plus minus} 0.02 oct, p = 0.09, Mann-Whitney U-test). These data have been added to the manuscript. Minor: Abstract 'within one week' - the context of this was unclear in the abstract, until the full methods section. Provide some context about the time frame in abstract. We have reworded this to a clearer statement. Intro 'Furthermore, various forms of ASD exhibit deficits which also underly FMRP regulation' - The meaning of this sentence is unclear. Changed to "Furthermore, various forms of ASD cause misexpression or malfunction of FMRP". Expand FMR1 at first use Done. Expand DGLalpha at first use Corrected. Method Justify why both sexes were used, and how the data were analyzed for sex. Because of the mosaicism issue in FXS, it is important to justify the use of both sexes in a study. We only used hemizygous male KO and homozygous female KO animals, not heterozygous female animals in our study. The possibility for mosaic expression of FMR1 should thus be minimal if present at all. To our knowledge, mosaic expression is not observed in full KO animals. In a recent study of PV neurons in somatosensory cortex, Kourdougli et al. (2023) found no differences between male and female FMR1 KO mice. We thus pooled the data from both sexes when analyzing differences between the genotypes, which we now state in the manuscript, along with the numbers of males and females. Page 5, methods 3rd line from bottom - 'mat'? Corrected. Page 7 12th line from bottom - check grammar Corrected. Page 8 - expand FRA at first use Done. Fmr1 KO mice are hyperactive - so does removing running related data lead to different amount of data being analyzed for WT vs KO mice? Despite the hyperactivity of FMR1 KO mice, the total running times of WT controls and KOs were 9.5 % {plus minus} 1.38 % and 10.9 % {plus minus} 3.43 % on average, with no significant difference (U-test, p = 1, Rebuttal figure 2). Thus, there shouldn't be a disproportional high loss of data in KO mice. Rebuttal figure 2: Total times running of WT and KO animals throughout all experiments. Page 17 - 'prominent represented' - grammar This sentence has been completely restructured. Page 17 top - statistics? Have been added, see response to major concern above. The discussion needs to take into account recent papers that show reduced long range and increased local connectivity in Fmr1 KO mice. Also papers on auditory system of Fmr1 KO rats are not referenced and are important comparative data sets. We have now included the works by Zhang et al. (2021), Haberl et al. (2015), Auerbach et al. (2021) and Engineer et al. (2014) into our discussion. Page 26 - Did Antoine et al., paper record from AC? We apologize for this mistake. We have removed the paper from this position, as recording were performed in somatosensory paper. References Auerbach BD, Manohar S, Radziwon K, Salvi R (2021) Auditory hypersensitivity and processing deficits in a rat model of fragile X syndrome. Neurobiology of Disease 161:105541. Bandyopadhyay S, Shamma SA, Kanold PO (2010) Dichotomy of functional organization in the mouse auditory cortex. Nat Neurosci 13:361-368. Bowen Z, Winkowski DE, Kanold PO (2020) Functional organization of mouse primary auditory cortex in adult C57BL/6 and F1 (CBAxC57) mice. Scientific Reports 10:10905. Engineer CT, Centanni TM, Im KW, Rahebi KC, Buell EP, Kilgard MP (2014) Degraded speech sound processing in a rat model of fragile X syndrome. Brain Res 1564:72-84. Gaucher Q, Panniello M, Ivanov AZ, Dahmen JC, King AJ, Walker KMM (2020) Complexity of frequency receptive fields predicts tonotopic variability across species. eLife 9:e53462. Gildin L, Rauti R, Vardi O, Kuznitsov-Yanovsky L, Maoz BM, Segal M, Ben-Yosef D (2022) Impaired Functional Connectivity Underlies Fragile X Syndrome. International journal of molecular sciences 23. Haberl MG, Zerbi V, Veltien A, Ginger M, Heerschap A, Frick A (2015) Structural-functional connectivity deficits of neocortical circuits in the Fmr1 (-/y) mouse model of autism. Sci Adv 1:e1500775. Issa John B, Haeffele Benjamin D, Agarwal A, Bergles Dwight E, Young Eric D, Yue David T (2014) Multiscale Optical Ca2+ Imaging of Tonal Organization in Mouse Auditory Cortex. Neuron 83:944-959. Kourdougli N, Suresh A, Liu B, Juarez P, Lin A, Chung DT, Graven Sams A, Gandal MJ, Martínez Cerdeño V, Buonomano DV, Hall BJ, Mombereau C, Portera-Cailliau C (2023) Improvement of sensory deficits in fragile X mice by increasing cortical interneuron activity after the critical period. Neuron 111:2863-2880.e2866. Kozono N, Okamura A, Honda S, Matsumoto M, Mihara T (2020) Gamma power abnormalities in a Fmr1-targeted transgenic rat model of fragile X syndrome. Sci Rep 10:18799. Rothschild G, Nelken I, Mizrahi A (2010) Functional organization and population dynamics in the mouse primary auditory cortex. Nat Neurosci 13:353-360. Ruby K, Falvey K, Kulesza RJ (2015) Abnormal neuronal morphology and neurochemistry in the auditory brainstem of Fmr1 knockout rats. Neuroscience 303:285-298. Wong H, Hooper AWM, Niibori Y, Lee SJ, Hategan LA, Zhang L, Karumuthil-Melethil S, Till SM, Kind PC, Danos O, Bruder JT, Hampson DR (2020) Sexually dimorphic patterns in electroencephalography power spectrum and autism-related behaviors in a rat model of fragile X syndrome. Neurobiol Dis 146:105118. Zhang Z, Gibson JR, Huber KM (2021) Experience-dependent weakening of callosal synaptic connections in the absence of postsynaptic FMRP. Elife 10
