# Supplementary figures and images for: Topography and Ensemble Activity in the Auditory Cortex of a Mouse Model of Fragile X Syndrome
Source: eNeuro. 2024 May 7;11(5):ENEURO.0396-23.2024. doi: 10.1523/ENEURO.0396-23.2024 (PMC11097631; doi:10.1523/ENEURO.0396-23.2024)

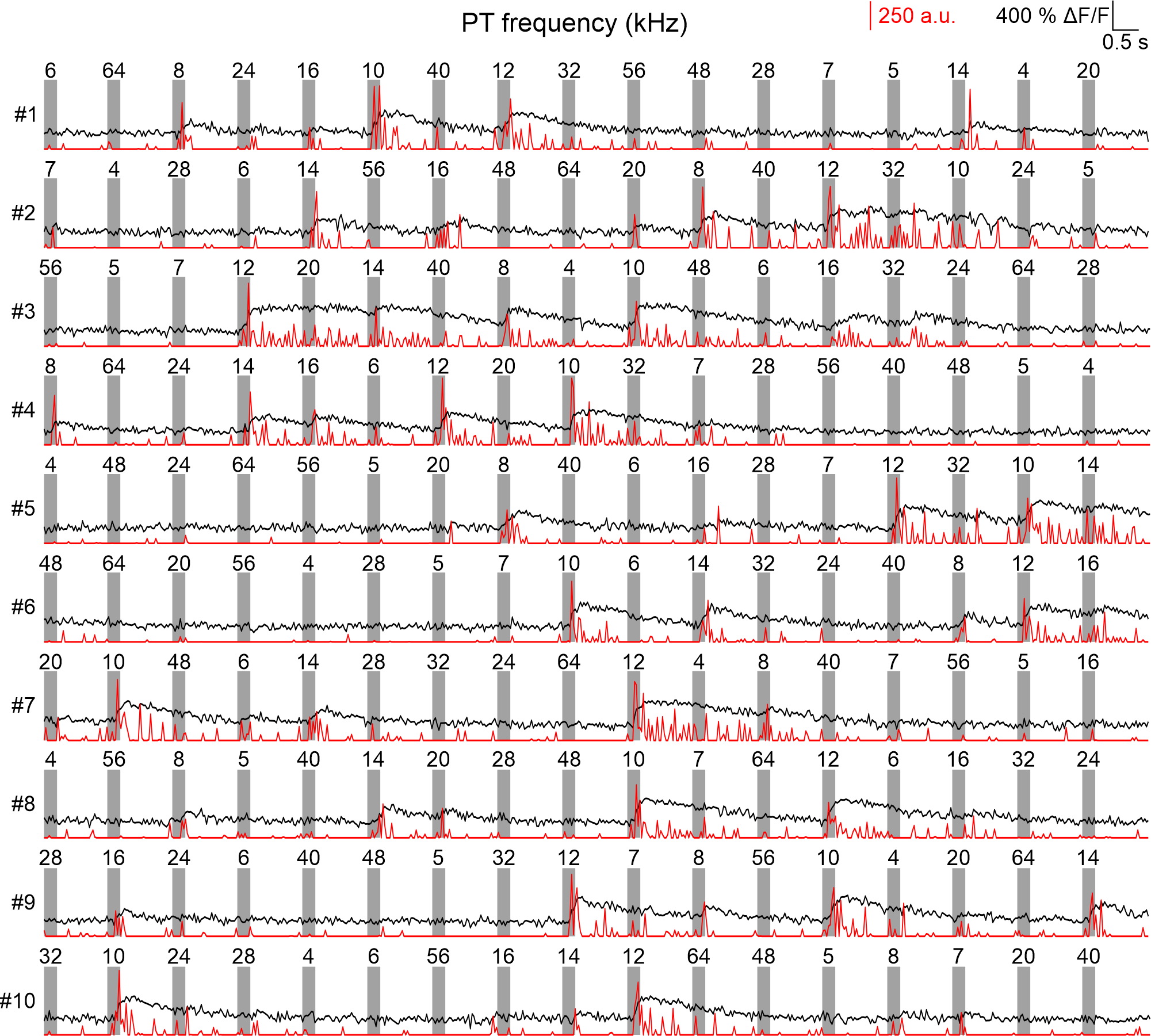

Supplement: Figure 1-1 — Response trace from an AC neuron in FMR1 KO. Uncut extracted fluorescent activity (ΔF/F, black trace) and corresponding deconvolution (red trace) during PT presentation at 60 dB SPL. Timings and length (250 ms) of presented PTs are illustrated by grey bars with the corresponding frequency depicted above. Each row denotes one repetition, containing each of the 17 PTs once. SLW performed experiments. Download Figure 1-1, TIF file. [file eneuro-11-ENEURO.0396-23.2024-s003.tif]

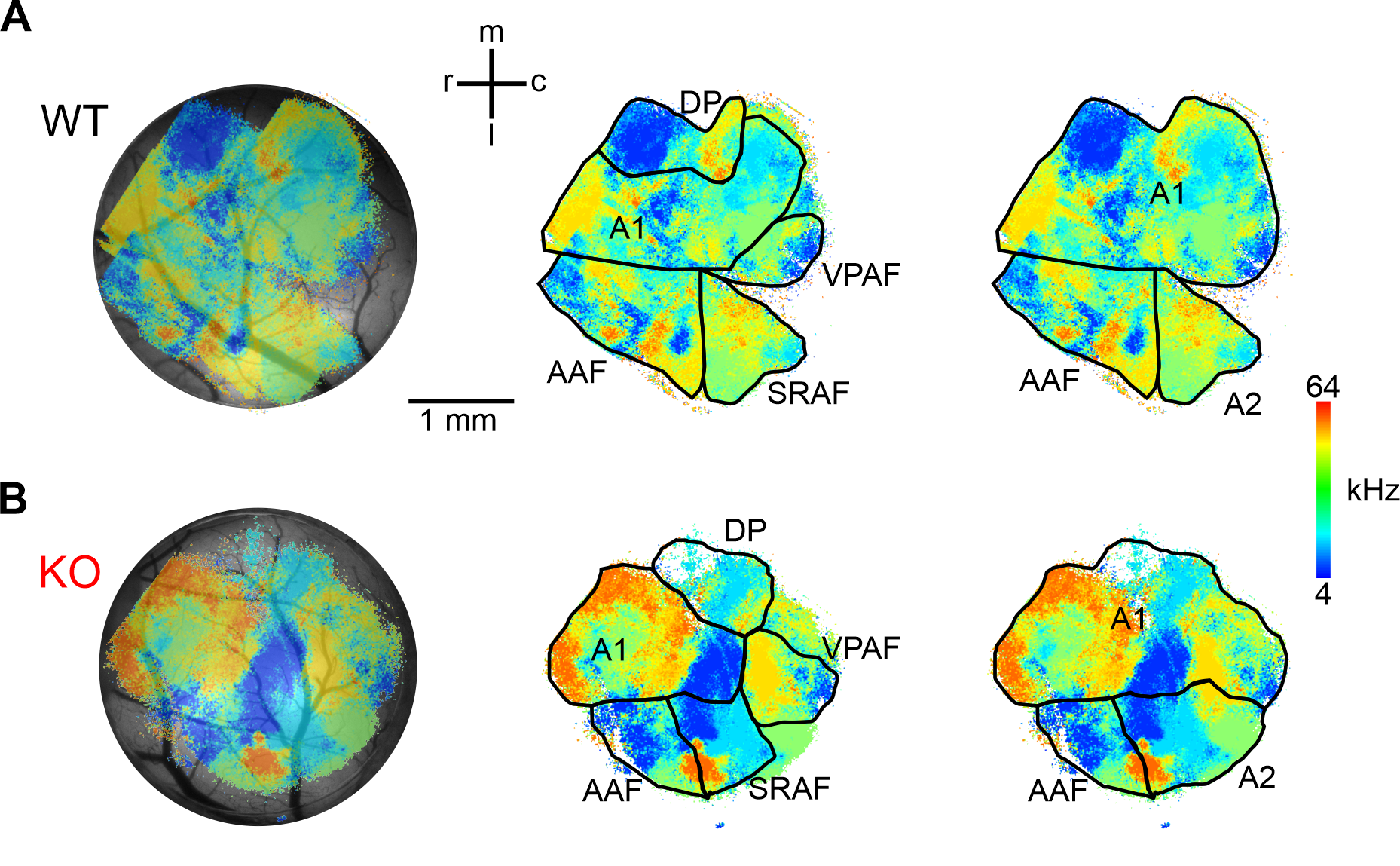

Supplement: Figure 1-2 — WF maps and subfield parcellation in FMR1 KO and littermate control. (A) Left: Cranial window with superimposed BF false-color map of a WT control. Middle: Same BF maps as left with borders of five subfields and nomenclature as in Romero et al. (2019). Right: Same map as middle and left but with borders drawn by merging DP and VPAF to A1 and adding tone responsive regions to the nearest subfield. SRAF was renamed as A2. (B) Same as (A) but for FMR1 KO. SLW performed experiments and analyzed data. Download Figure 1-2, TIF file. [file eneuro-11-ENEURO.0396-23.2024-s004.tif]

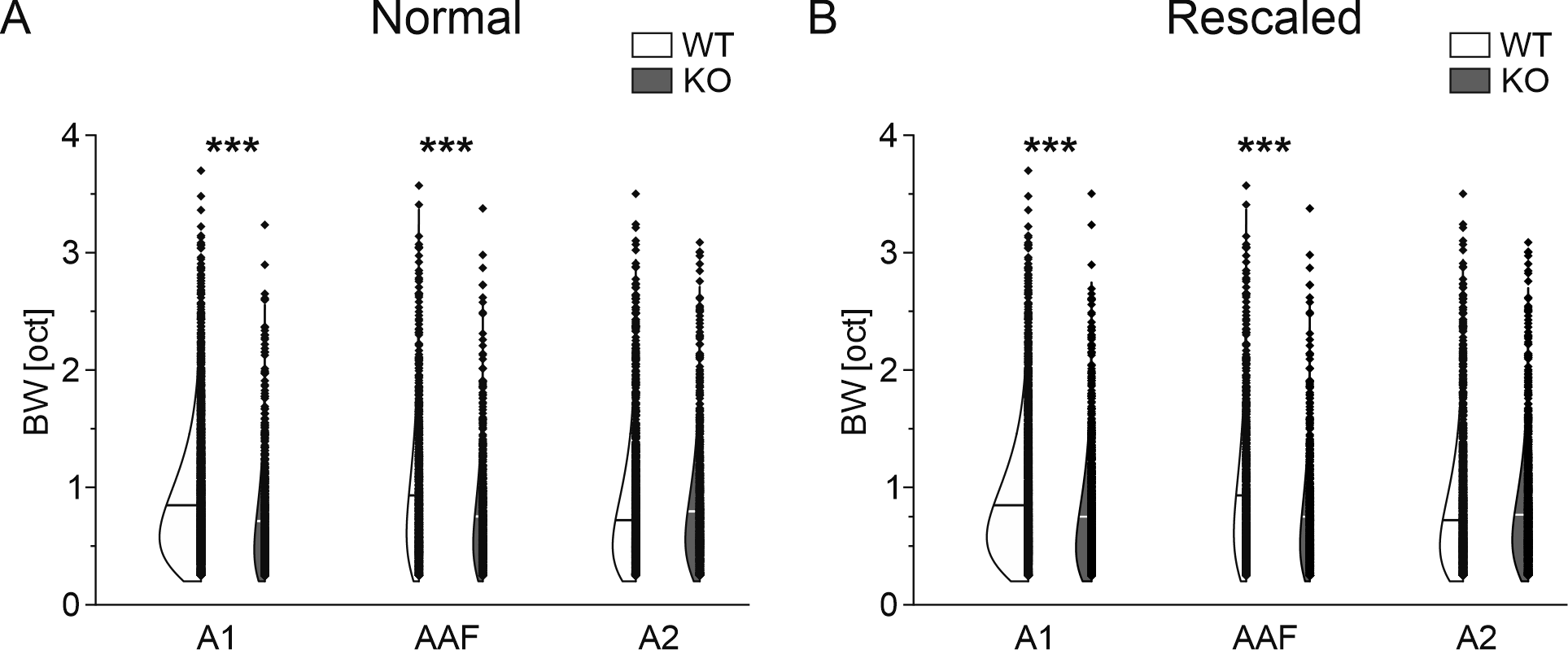

Supplement: Figure 1-3 — BW analysis after rescaling of traces in KO animals. (A) Tuning BW of single-peak neurons by subfield. (B) Same as (A), but after rescaling of traces obtained from KO animals. SLW performed experiments and analyzed data. Download Figure 1-3, TIF file. [file eneuro-11-ENEURO.0396-23.2024-s005.tif]

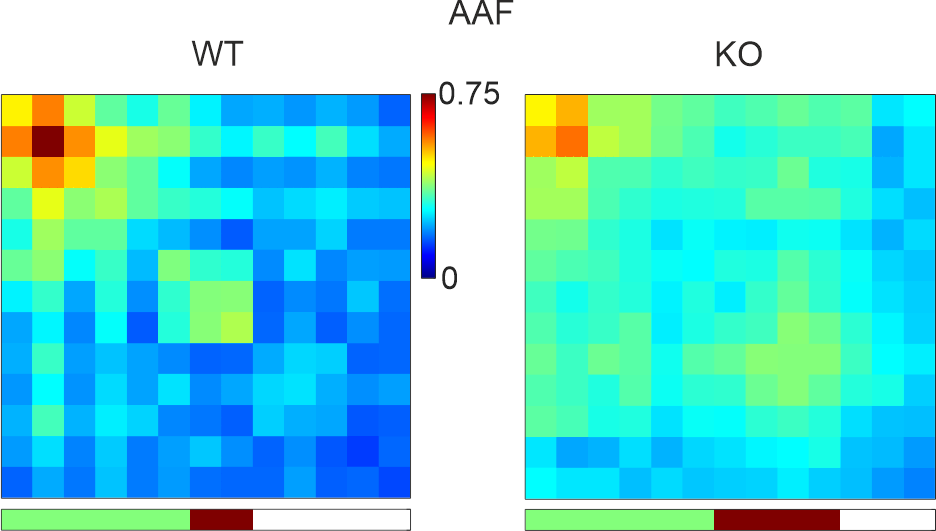

Supplement: Figure 5-1 — Correlation matrices of 13 complex sound-evoked patterns after hierarchical clustering, recorded in AAF. Color code depicts correlation value. Vertical color bar at the bottom depicts clusters, blank stripes correspond to sounds that are not part of clusters. The diagonal depicts mean correlation across repetitions, thus showing reliability of the network. Download Figure 5-1, TIF file. [file eneuro-11-ENEURO.0396-23.2024-s006.tif]

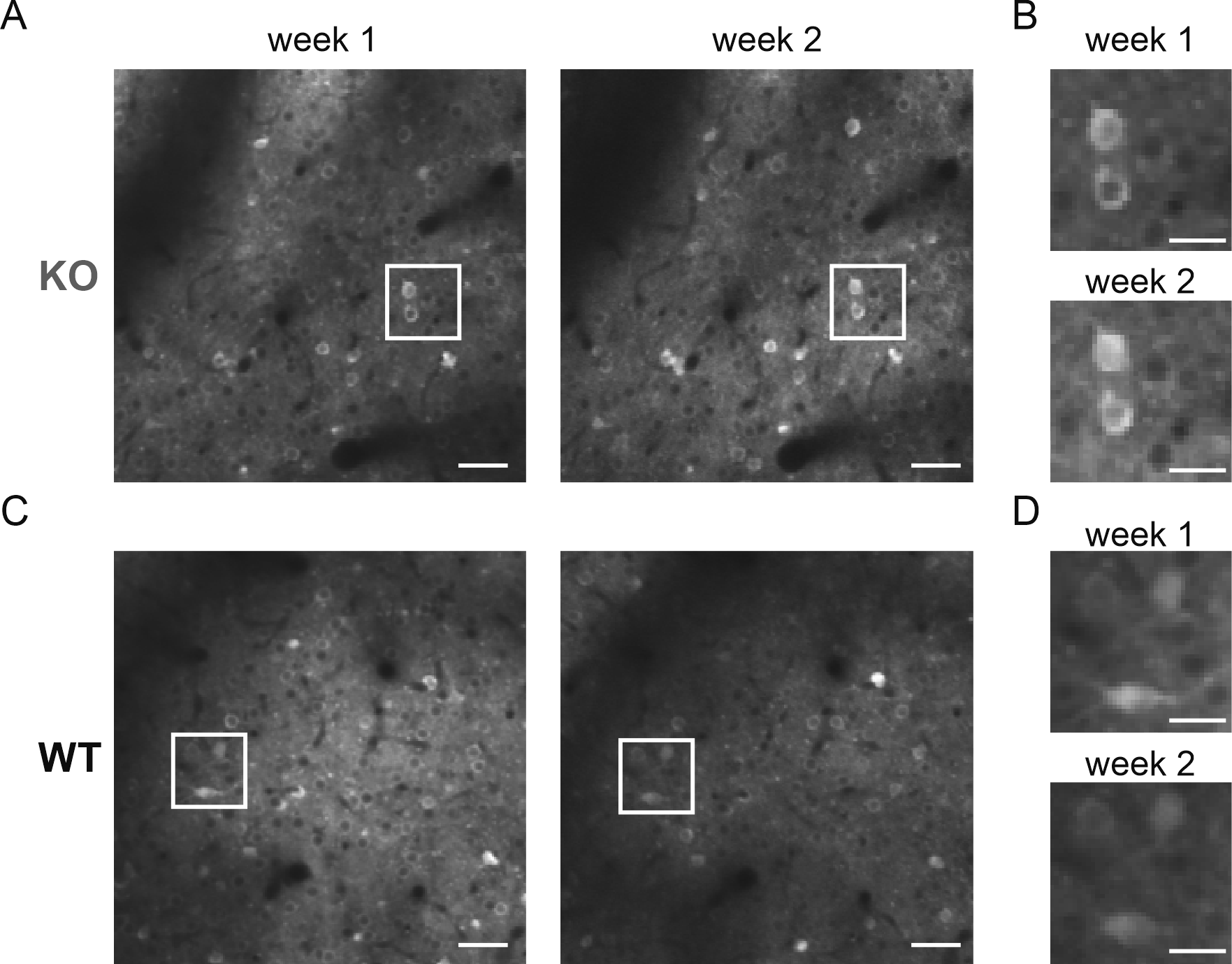

Supplement: Figure 6-1 — Neurons can be identified across experimental days. (A) Example section of a FOV in FMR1 KO in week 1 and the same section in week 2. Scale bar: 50 µm. (B) Magnified sections from the rectangles in (A) in week 1 and 2, respectively. Scale bar: 10 µm. (C) Same as (A), but for an example obtained from a WT animal. (D) Same as (B) but showing the magnifications from (C). Download Figure 6-1, TIF file. [file eneuro-11-ENEURO.0396-23.2024-s007.tif]

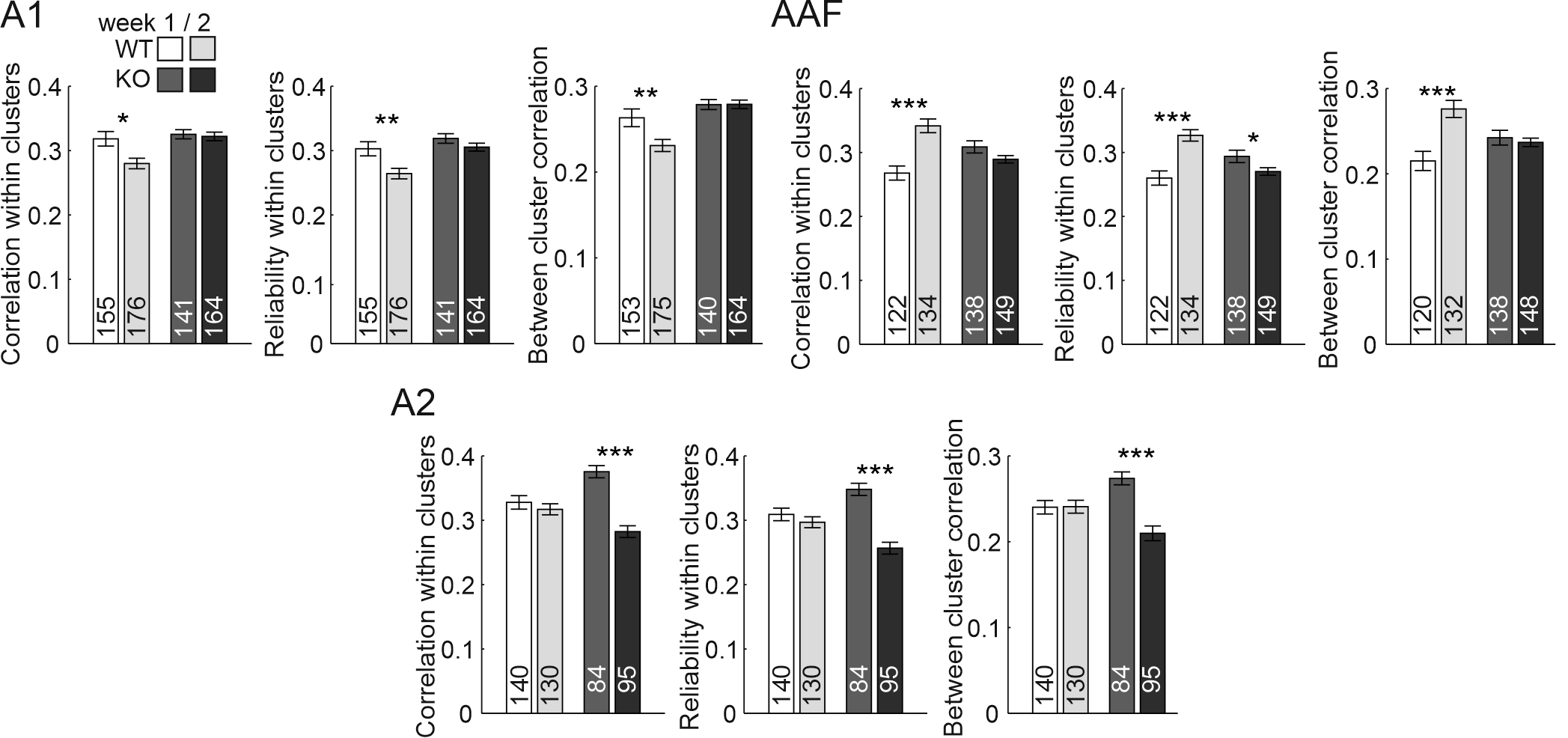

Supplement: Figure 6-2 — Alteration in correlation values between sound-evoked activity patterns across one week. 64 sound-evoked patterns (17 patterns for each of PTs, AM tones with 20Hz modulation, AM tones with 40 Hz modulation, and 13 patterns for complex sounds) were analyzed. Numbers in bars depict n-number (sound clusters). SLW performed experiments. JJH analyzed data. Download Figure 6-2, TIF file. [file eneuro-11-ENEURO.0396-23.2024-s008.tif]
