# Supplementary material for: Topography and Ensemble Activity in the Auditory Cortex of a Mouse Model of Fragile X Syndrome
Source: eNeuro. 2024 May 7;11(5):ENEURO.0396-23.2024. doi: 10.1523/ENEURO.0396-23.2024 (PMC11097631; doi:10.1523/ENEURO.0396-23.2024)
Supplement: Table 1-1 — Statistical analysis of tuning types per FOV. Only FOVs with at least 100 neurons per subfield were included in the analysis. Compared are values obtained from FMR1 KO mice and WT controls. U-test = Mann-Whitney U test. Download Table 1-1, DOCX file. [file eneuro-11-ENEURO.0396-23.2024-s009.docx]

|  | Single-peak | Double-peak | irregular |
| --- | --- | --- | --- |
| **A1** |  |  |  |
| WT | 19% ± 2.7% | 3% ± 0.4% | 78% ± 3.0% |
| KO | 13% ± 2.6% | 2% ± 0.3% | 85% ± 2.8% |
| n(WT) | 40 | | |
| n(KO) | 19 | | |
| *p*-value | 0.403 | 0.351 | 0.368 |
| Stat. test | U-test | U-test | U-test |
| **AAF** |  |  |  |
| WT | 18% ± 5.3% | 3% ± 0.7% | 79% ± 5.8% |
| KO | 14% ± 2.1% | 2% ± 0.7% | 84% ± 2.6% |
| n(WT) | 11 | | |
| n(KO) | 18 | | |
| *p*-value | 0.574 | 0.574 | 0.669 |
| Stat. test | U-test | U-test | U-test |
| **A2** |  |  |  |
| WT | 30% ± 3.6% | 7% ± 0.7% | 63% ± 3.5% |
| KO | 30% ± 3.2% | 3% ± 0.5% | 67% ± 3.4% |
| n(WT) | 12 | | |
| n(KO) | 10 | | |
| *p*-value | 0.817 | 0.008 | 0.489 |
| Stat. test | U-test | U-test | U-test |
