# Supplementary material for: Topography and Ensemble Activity in the Auditory Cortex of a Mouse Model of Fragile X Syndrome
Source: eNeuro. 2024 May 7;11(5):ENEURO.0396-23.2024. doi: 10.1523/ENEURO.0396-23.2024 (PMC11097631; doi:10.1523/ENEURO.0396-23.2024)
Supplement: Table 4-1 — Statistical analysis of AC ensemble activity in response to 17 PTs. Compared are values obtained from FMR1 KO mice and WT controls. s. = sounds, c. = clusters, corr. = correlation, rel. = reliability, T-test2 = unpaired t-test, U-test = Mann-Whitney U test. Download Table 4-1, DOCX file. [file eneuro-11-ENEURO.0396-23.2024-s010.docx]

|  | No. of c. | S. per c. | Fraction of clustered s. | Corr. within c. | Rel. within c. | Corr. between c. |
| --- | --- | --- | --- | --- | --- | --- |
| **A1** |  |  |  |  |  |  |
| WT | 2.38 ± 0.14 | 5.56 ± 0.34 | 0.78 ± 0.02 | 0.34 ± 0.01 | 0.33 ± 0.01 | 0.27 ± 0.01 |
| KO | 2.3 ± 0.15 | 5.43 ± 0.4 | 0.73 ± 0.03 | 0.3 ± 0.01 | 0.29 ± 0.01 | 0.26 ± 0.01 |
| n(WT) | 74 | 176 | 74 | 176 | 176 | 154 |
| n(KO) | 50 | 115 | 50 | 115 | 115 | 103 |
| *p*-value | 0.96419 | 0.99364 | 0.17294 | 0.010052 | 0.0089266 | 0.60326 |
| Stat. test | U-test | U-test | U-test | T-test2 | T-test2 | U-test |
| **AAF** |  |  |  |  |  |  |
| WT | 2.41 ± 0.18 | 5.3 ± 0.4 | 0.75 ± 0.03 | 0.29 ± 0.01 | 0.26 ± 0.01 | 0.22 ± 0.01 |
| KO | 2.07 ± 0.15 | 5.88 ± 0.54 | 0.72 ± 0.03 | 0.28 ± 0.01 | 0.28 ± 0.01 | 0.25 ± 0.01 |
| n(WT) | 44 | 106 | 44 | 106 | 106 | 96 |
| n(KO) | 44 | 91 | 44 | 91 | 91 | 75 |
| *p*-value | 0.18469 | 0.8479 | 0.70874 | 0.83372 | 0.46981 | 0.099738 |
| Stat. test | U-test | U-test | U-test | T-test2 | T-test2 | T-test2 |
| **A2** |  |  |  |  |  |  |
| WT | 3.09 ± 0.2 | 4.26 ± 0.28 | 0.77 ± 0.03 | 0.36 ± 0.01 | 0.32 ± 0.01 | 0.28 ± 0.01 |
| KO | 2.78 ± 0.18 | 4.88 ± 0.39 | 0.8 ± 0.03 | 0.36 ± 0.01 | 0.34 ± 0.01 | 0.28 ± 0.01 |
| n(WT) | 43 | 133 | 43 | 133 | 133 | 127 |
| n(KO) | 27 | 75 | 27 | 75 | 75 | 73 |
| *p*-value | 0.28446 | 0.082208 | 0.59399 | 0.93232 | 0.19668 | 0.71859 |
| Stat. test | T-test2 | U-test | T-test2 | T-test2 | T-test2 | U-test |
