# Supplementary material for: Topography and Ensemble Activity in the Auditory Cortex of a Mouse Model of Fragile X Syndrome
Source: eNeuro. 2024 May 7;11(5):ENEURO.0396-23.2024. doi: 10.1523/ENEURO.0396-23.2024 (PMC11097631; doi:10.1523/ENEURO.0396-23.2024)
Supplement: Table 4-2 — Statistical analysis of AC ensemble activity in A1 in response to 17 PTs played at different SPLs. Compared are values obtained from FMR1 KO mice and WT controls. s. = sounds, c. = clusters, corr. = correlation, rel. = reliability, T-test2 = unpaired t-test, U-test = Mann-Whitney U test. Download Table 4-2, DOCX file. [file eneuro-11-ENEURO.0396-23.2024-s011.docx]

|  | No. of c. | S. per c. | Fraction of clustered s. | Corr. within c. | Rel. within c. | Corr. between c. |
| --- | --- | --- | --- | --- | --- | --- |
| **30 dB** |  |  |  |  |  |  |
| WT | 1.93 ± 0.11 | 6.91 ± 0.43 | 0.79 ± 0.02 | 0.29 ± 0.01 | 0.28 ± 0.01 | 0.26 ± 0.01 |
| KO | 2.16 ± 0.13 | 5.23 ± 0.43 | 0.66 ± 0.03 | 0.29 ± 0.01 | 0.27 ± 0.01 | 0.25 ± 0.01 |
| n(WT) | 89 | 172 | 89 | 172 | 172 | 134 |
| n(KO) | 50 | 108 | 50 | 108 | 108 | 97 |
| *p*-value | 0.097564 | 0.073261 | 0.00099209 | 0.87809 | 0.64757 | 0.65572 |
| Stat. test | U-test | U-test | U-test | T-test2 | U-test | T-test2 |
| **40 dB** |  |  |  |  |  |  |
| WT | 2.01 ± 0.11 | 6.47 ± 0.4 | 0.77 ± 0.02 | 0.28 ± 0.01 | 0.26 ± 0.01 | 0.24 ± 0.01 |
| KO | 2.36 ± 0.21 | 5.4 ± 0.48 | 0.75 ± 0.03 | 0.29 ± 0.01 | 0.27 ± 0.01 | 0.26 ± 0.01 |
| n(WT) | 93 | 187 | 93 | 187 | 187 | 147 |
| n(KO) | 47 | 111 | 47 | 111 | 111 | 93 |
| *p*-value | 0.23895 | 0.071629 | 0.61591 | 0.076708 | 0.70414 | 0.27134 |
| Stat. test | U-test | U-test | U-test | U-test | T-test2 | T-test2 |
| **50 dB** |  |  |  |  |  |  |
| WT | 2.11 ± 0.12 | 6.39 ± 0.39 | 0.79 ± 0.02 | 0.29 ± 0.01 | 0.28 ± 0.01 | 0.25 ± 0.01 |
| KO | 2.34 ± 0.16 | 5.17 ± 0.42 | 0.71 ± 0.03 | 0.29 ± 0.01 | 0.27 ± 0.01 | 0.25 ± 0.01 |
| n(WT) | 88 | 186 | 88 | 186 | 186 | 152 |
| n(KO) | 50 | 117 | 50 | 117 | 117 | 104 |
| *p*-value | 0.15883 | 0.11572 | 0.038896 | 0.52717 | 0.22293 | 0.62038 |
| Stat. test | U-test | U-test | U-test | U-test | T-test2 | T-test2 |
| **60 dB** |  |  |  |  |  |  |
| WT | 2.28 ± 0.14 | 5.91 ± 0.35 | 0.79 ± 0.02 | 0.3 ± 0.01 | 0.29 ± 0.01 | 0.25 ± 0.01 |
| KO | 2 ± 0.12 | 6.34 ± 0.47 | 0.75 ± 0.03 | 0.29 ± 0.01 | 0.28 ± 0.01 | 0.26 ± 0.01 |
| n(WT) | 87 | 198 | 87 | 198 | 198 | 167 |
| n(KO) | 52 | 104 | 52 | 104 | 104 | 88 |
| *p*-value | 0.42221 | 0.26254 | 0.20526 | 0.28227 | 0.21382 | 0.45378 |
| Stat. test | U-test | U-test | U-test | T-test2 | T-test2 | T-test2 |
| **70 dB** |  |  |  |  |  |  |
| WT | 2.28 ± 0.13 | 5.88 ± 0.34 | 0.79 ± 0.02 | 0.32 ± 0.01 | 0.32 ± 0.01 | 0.26 ± 0.01 |
| KO | 2.53 ± 0.19 | 4.89 ± 0.38 | 0.73 ± 0.03 | 0.31 ± 0.01 | 0.29 ± 0.01 | 0.26 ± 0.01 |
| n(WT) | 90 | 205 | 90 | 205 | 205 | 175 |
| n(KO) | 51 | 129 | 51 | 129 | 129 | 116 |
| *p*-value | 0.27743 | 0.028891 | 0.080412 | 0.30906 | 0.020961 | 0.83359 |
| Stat. test | U-test | U-test | U-test | U-test | T-test2 | T-test2 |
