# Supplementary material for: Topography and Ensemble Activity in the Auditory Cortex of a Mouse Model of Fragile X Syndrome
Source: eNeuro. 2024 May 7;11(5):ENEURO.0396-23.2024. doi: 10.1523/ENEURO.0396-23.2024 (PMC11097631; doi:10.1523/ENEURO.0396-23.2024)
Supplement: Table 5-1 — Statistical analysis of AC ensemble activity in response to 13 complex sounds. Compared are values obtained from FMR1 KO mice and WT controls. s. = sounds, c. = clusters, corr. = correlation, rel. = reliability, T-test2 = unpaired t-test, U-test = Mann-Whitney U test. Download Table 5-1, DOCX file. [file eneuro-11-ENEURO.0396-23.2024-s012.docx]

|  | No. of c. | S. per c. | Fraction of clustered s. | Corr. within c. | Rel. within c. | Corr. between c. |
| --- | --- | --- | --- | --- | --- | --- |
| **A1** |  |  |  |  |  |  |
| WT | 2.07 ± 0.12 | 5.05 ± 0.32 | 0.8 ± 0.02 | 0.4 ± 0.01 | 0.43 ± 0.01 | 0.33 ± 0.01 |
| KO | 1.98 ± 0.14 | 5.4 ± 0.41 | 0.82 ± 0.02 | 0.37 ± 0.01 | 0.38 ± 0.01 | 0.31 ± 0.01 |
| n(WT) | 59 | 122 | 59 | 122 | 122 | 102 |
| n(KO) | 44 | 87 | 44 | 87 | 87 | 71 |
| *p*-value | 0.57943 | 0.83274 | 0.37768 | 0.036743 | 0.004583 | 0.13222 |
| Stat. test | U-test | U-test | U-test | T-test2 | T-test2 | T-test2 |
| **AAF** |  |  |  |  |  |  |
| WT | 1.95 ± 0.17 | 5.68 ± 0.46 | 0.85 ± 0.02 | 0.35 ± 0.02 | 0.37 ± 0.02 | 0.23 ± 0.01 |
| KO | 2.15 ± 0.15 | 4.77 ± 0.38 | 0.79 ± 0.03 | 0.35 ± 0.01 | 0.37 ± 0.01 | 0.29 ± 0.01 |
| n(WT) | 37 | 72 | 37 | 72 | 72 | 56 |
| n(KO) | 33 | 71 | 33 | 71 | 71 | 64 |
| *p*-value | 0.2192 | 0.18564 | 0.02971 | 0.87075 | 0.92162 | 0.0013272 |
| Stat. test | U-test | U-test | U-test | T-test2 | T-test2 | T-test2 |
| **A2** |  |  |  |  |  |  |
| WT | 2.76 ± 0.16 | 3.83 ± 0.25 | 0.81 ± 0.02 | 0.4 ± 0.01 | 0.42 ± 0.01 | 0.3 ± 0.01 |
| KO | 2.83 ± 0.19 | 3.73 ± 0.25 | 0.81 ± 0.04 | 0.44 ± 0.02 | 0.47 ± 0.01 | 0.33 ± 0.01 |
| n(WT) | 37 | 102 | 37 | 102 | 102 | 99 |
| n(KO) | 18 | 51 | 18 | 51 | 51 | 51 |
| *p*-value | 0.80579 | 0.35494 | 0.89062 | 0.035313 | 0.015896 | 0.18161 |
| Stat. test | U-test | U-test | U-test | T-test2 | T-test2 | T-test2 |
