# Supplementary material for: Topography and Ensemble Activity in the Auditory Cortex of a Mouse Model of Fragile X Syndrome
Source: eNeuro. 2024 May 7;11(5):ENEURO.0396-23.2024. doi: 10.1523/ENEURO.0396-23.2024 (PMC11097631; doi:10.1523/ENEURO.0396-23.2024)
Supplement: Table 6-1 — Statistical analysis of AC ensemble activity in response to 17 PTs, 34 AM-modulated tones and 13 complex sounds, with data collected in the first week of experiments. Compared are values obtained from FMR1 KO mice and WT controls. s. = sounds, c. = clusters, corr. = correlation, rel. = reliability, T-test2 = unpaired t-test, U-test = Mann-Whitney U test. Download Table 6-1, DOCX file. [file eneuro-11-ENEURO.0396-23.2024-s013.docx]

|  | No. of c. | S. per c. | Fraction of clustered s. | Corr. within c. | Rel. within c. | Corr. between c. |
| --- | --- | --- | --- | --- | --- | --- |
| **A1** |  |  |  |  |  |  |
| WT | 6.6 ± 0.5 | 5.99 ± 0.49 | 0.62 ± 0.03 | 0.34 ± 0.01 | 0.32 ± 0.01 | 0.26 ± 0.01 |
| KO | 6.06 ± 0.59 | 6.06 ± 0.57 | 0.57 ± 0.03 | 0.3 ± 0.01 | 0.29 ± 0.01 | 0.25 ± 0.01 |
| n(WT) | 48 | 317 | 48 | 317 | 317 | 314 |
| n(KO) | 36 | 218 | 36 | 218 | 218 | 216 |
| *p*-value | 0.48107 | 0.88432 | 0.30612 | 0.066373 | 0.0083483 | 0.078459 |
| Stat. test | T-test2 | U-test | T-test2 | U-test | T-test2 | U-test |
| **AAF** |  |  |  |  |  |  |
| WT | 7.06 ± 0.72 | 6.15 ± 0.56 | 0.68 ± 0.03 | 0.28 ± 0.01 | 0.26 ± 0.01 | 0.19 ± 0.01 |
| KO | 5.9 ± 0.55 | 5.88 ± 0.53 | 0.54 ± 0.03 | 0.3 ± 0.01 | 0.29 ± 0.01 | 0.24 ± 0.01 |
| n(WT) | 36 | 254 | 36 | 254 | 254 | 252 |
| n(KO) | 30 | 177 | 30 | 177 | 177 | 176 |
| *p*-value | 0.22128 | 0.87701 | 0.0037406 | 0.016959 | 0.0014481 | 3.546e-06 |
| Stat. test | T-test2 | U-test | T-test2 | T-test2 | T-test2 | T-test2 |
| **A2** |  |  |  |  |  |  |
| WT | 8.45 ± 0.57 | 5.06 ± 0.26 | 0.67 ± 0.02 | 0.35 ± 0.01 | 0.32 ± 0.01 | 0.25 ± 0.01 |
| KO | 8.07 ± 0.89 | 5.43 ± 0.54 | 0.68 ± 0.04 | 0.37 ± 0.01 | 0.34 ± 0.01 | 0.27 ± 0.01 |
| n(WT) | 33 | 279 | 33 | 279 | 279 | 279 |
| n(KO) | 15 | 121 | 15 | 121 | 121 | 121 |
| *p*-value | 0.70873 | 0.32988 | 0.69295 | 0.09135 | 0.085338 | 0.00035289 |
| Stat. test | T-test2 | U-test | T-test2 | T-test2 | T-test2 | U-test |
