# Supplementary material for: Topography and Ensemble Activity in the Auditory Cortex of a Mouse Model of Fragile X Syndrome
Source: eNeuro. 2024 May 7;11(5):ENEURO.0396-23.2024. doi: 10.1523/ENEURO.0396-23.2024 (PMC11097631; doi:10.1523/ENEURO.0396-23.2024)
Supplement: Table 6-2 — Statistical analysis of AC ensemble activity in response to 17 PTs, 34 AM-modulated tones and 13 complex sounds, with data collected in the second week of experiments. Compared are values obtained from FMR1 KO mice and WT controls. s. = sounds, c. = clusters, corr. = correlation, rel. = reliability, T-test2 = unpaired t-test, U-test = Mann-Whitney U test. Download Table 6-2, DOCX file. [file eneuro-11-ENEURO.0396-23.2024-s014.docx]

|  | No. of c. | S. per c. | Fraction of clustered s. | Corr. within c. | Rel. within c. | Corr. between c. |
| --- | --- | --- | --- | --- | --- | --- |
| **A1** |  |  |  |  |  |  |
| WT | 6.61 ± 0.59 | 5.33 ± 0.43 | 0.55 ± 0.03 | 0.29 ± 0.01 | 0.27 ± 0.01 | 0.24 ± 0.01 |
| KO | 7.84 ± 0.68 | 4.81 ± 0.3 | 0.59 ± 0.02 | 0.31 ± 0 | 0.29 ± 0 | 0.26 ± 0 |
| n(WT) | 41 | 271 | 41 | 271 | 271 | 270 |
| n(KO) | 38 | 298 | 38 | 298 | 298 | 298 |
| *p*-value | 0.17329 | 0.74398 | 0.31256 | 0.00018386 | 9.8227e-05 | 1.43e-08 |
| Stat. test | T-test2 | U-test | T-test2 | U-test | U-test | U-test |
| **AAF** |  |  |  |  |  |  |
| WT | 6.75 ± 0.67 | 5.94 ± 0.59 | 0.63 ± 0.03 | 0.32 ± 0.01 | 0.29 ± 0.01 | 0.25 ± 0.01 |
| KO | 6.7 ± 0.72 | 5.91 ± 0.59 | 0.62 ± 0.03 | 0.3 ± 0.005 | 0.28 ± 0.005 | 0.25 ± 0.004 |
| n(WT) | 32 | 216 | 32 | 216 | 216 | 213 |
| n(KO) | 33 | 221 | 33 | 221 | 221 | 220 |
| *p*-value | 0.95732 | 0.26743 | 0.86648 | 0.051438 | 0.16761 | 0.40525 |
| Stat. test | T-test2 | U-test | T-test2 | T-test2 | U-test | U-test |
| **A2** |  |  |  |  |  |  |
| WT | 6.96 ± 0.59 | 6.49 ± 0.48 | 0.71 ± 0.03 | 0.32 ± 0.01 | 0.3 ± 0.01 | 0.24 ± 0.01 |
| KO | 8.89 ± 0.79 | 5.13 ± 0.32 | 0.71 ± 0.03 | 0.3 ± 0.01 | 0.29 ± 0.01 | 0.23 ± 0.01 |
| n(WT) | 25 | 174 | 25 | 174 | 174 | 174 |
| n(KO) | 19 | 169 | 19 | 169 | 169 | 169 |
| *p*-value | 0.051585 | 0.13694 | 0.84976 | 0.039255 | 0.58642 | 0.82035 |
| Stat. test | T-test2 | U-test | T-test2 | T-test2 | U-test | U-test |
