# Supplementary material for: Topography and Ensemble Activity in the Auditory Cortex of a Mouse Model of Fragile X Syndrome
Source: eNeuro. 2024 May 7;11(5):ENEURO.0396-23.2024. doi: 10.1523/ENEURO.0396-23.2024 (PMC11097631; doi:10.1523/ENEURO.0396-23.2024)
Supplement: Table 6-3 — Statistical analysis of AC ensemble activity in response to 17 PTs, 34 AM-modulated tones and 13 complex sounds, comparing values obtained from WT mice across one week. s. = sounds, c. = clusters, corr. = correlation, rel. = reliability, T-test = paired t-test, T-test2 = unpaired t-test, U-test = Mann-Whitney U test. Download Table 6-3, DOCX file. [file eneuro-11-ENEURO.0396-23.2024-s015.docx]

|  | No. of c. | S. per c. | Fraction of clustered s. | Corr. within c. | Rel. within c. | Corr. between c. |
| --- | --- | --- | --- | --- | --- | --- |
| **A1** |  |  |  |  |  |  |
| Week 1 | 6.2 ± 0.7 | 6.23 ± 0.79 | 0.6 ± 0.04 | 0.32 ± 0.01 | 0.3 ± 0.01 | 0.26 ± 0.01 |
| Week 2 | 7.04 ± 0.85 | 5.56 ± 0.61 | 0.61 ± 0.03 | 0.28 ± 0.01 | 0.26 ± 0.01 | 0.23 ± 0.01 |
| n(week 1) | 25 | 155 | 25 | 155 | 155 | 153 |
| n(week 2) | 25 | 176 | 25 | 176 | 176 | 175 |
| *p*-value | 0.40972 | 0.1165 | 0.88972 | 0.01456 | 0.0094254 | 0.0059629 |
| Stat. test | T-test | U-test | T-test | U-test | U-test | U-test |
| **AAF** |  |  |  |  |  |  |
| Week 1 | 5.81 ± 0.92 | 7.36 ± 0.98 | 0.67 ± 0.04 | 0.27 ± 0.01 | 0.26 ± 0.01 | 0.22 ± 0.01 |
| Week 2 | 6.38 ± 0.71 | 6.07 ± 0.74 | 0.61 ± 0.04 | 0.34 ± 0.01 | 0.33 ± 0.01 | 0.28 ± 0.01 |
| n(week 1) | 21 | 122 | 21 | 122 | 122 | 120 |
| n(week 2) | 21 | 134 | 21 | 134 | 134 | 132 |
| *p*-value | 0.61015 | 0.86483 | 0.33426 | 2.1745e-05 | 0.00014768 | 6.6855e-05 |
| Stat. test | T-test | U-test | T-test | U-test | U-test | T-test2 |
| **A2** |  |  |  |  |  |  |
| Week 1 | 8.24 ± 0.81 | 5.12 ± 0.4 | 0.66 ± 0.03 | 0.33 ± 0.01 | 0.31 ± 0.01 | 0.24 ± 0.01 |
| Week 2 | 7.65 ± 0.75 | 5.92 ± 0.52 | 0.71 ± 0.03 | 0.32 ± 0.01 | 0.3 ± 0.01 | 0.24 ± 0.01 |
| n(week 1) | 17 | 140 | 17 | 140 | 140 | 140 |
| n(week 2) | 17 | 130 | 17 | 130 | 130 | 130 |
| *p*-value | 0.58447 | 0.49246 | 0.29087 | 0.4283 | 0.34835 | 0.98444 |
| Stat. test | T-test | U-test | T-test | T-test2 | T-test2 | U-test |
