# Supplementary material for: Topography and Ensemble Activity in the Auditory Cortex of a Mouse Model of Fragile X Syndrome
Source: eNeuro. 2024 May 7;11(5):ENEURO.0396-23.2024. doi: 10.1523/ENEURO.0396-23.2024 (PMC11097631; doi:10.1523/ENEURO.0396-23.2024)
Supplement: Table 6-4 — Statistical analysis of AC ensemble activity in response to 17 PTs, 34 AM-modulated tones and 13 complex sounds, comparing values obtained from FMR1 KO mice across one week. s. = sounds, c. = clusters, corr. = correlation, rel. = reliability, T-test = paired t-test, T-test2 = unpaired t-test, U-test = Mann-Whitney U test. Download Table 6-4, DOCX file. [file eneuro-11-ENEURO.0396-23.2024-s016.docx]

|  | No. of c. | S. per c. | Fraction of clustered s. | Corr. within c. | Rel. within c. | Corr. between c. |
| --- | --- | --- | --- | --- | --- | --- |
| **A1** |  |  |  |  |  |  |
| Week 1 | 5.64 ± 0.58 | 6.35 ± 0.66 | 0.56 ± 0.03 | 0.33 ± 0.01 | 0.32 ± 0.01 | 0.28 ± 0.01 |
| Week 2 | 6.56 ± 0.74 | 5.35 ± 0.45 | 0.55 ± 0.03 | 0.32 ± 0.01 | 0.31 ± 0.01 | 0.28 ± 0.005 |
| n(week 1) | 25 | 141 | 25 | 141 | 141 | 140 |
| n(week 2) | 25 | 164 | 25 | 164 | 164 | 164 |
| *p*-value | 0.13287 | 0.22639 | 0.78122 | 0.73404 | 0.16109 | 0.96685 |
| Stat. test | T-test | U-test | T-test | T-test2 | T-test2 | T-test2 |
| **AAF** |  |  |  |  |  |  |
| Week 1 | 5.75 ± 0.6 | 5.83 ± 0.51 | 0.52 ± 0.04 | 0.31 ± 0.01 | 0.29 ± 0.01 | 0.24 ± 0.01 |
| Week 2 | 6.21 ± 0.83 | 6.38 ± 0.74 | 0.62 ± 0.04 | 0.29 ± 0.01 | 0.27 ± 0.01 | 0.24 ± 0.005 |
| n(week 1) | 24 | 138 | 24 | 138 | 138 | 138 |
| n(week 2) | 24 | 149 | 24 | 149 | 149 | 148 |
| *p*-value | 0.59994 | 0.71868 | 0.093646 | 0.078477 | 0.034893 | 0.57931 |
| Stat. test | T-test | U-test | T-test | T-test2 | T-test2 | T-test2 |
| **A2** |  |  |  |  |  |  |
| Week 1 | 7.64 ± 0.98 | 5.64 ± 0.62 | 0.67 ± 0.04 | 0.38 ± 0.01 | 0.35 ± 0.01 | 0.27 ± 0.01 |
| Week 2 | 8.64 ± 0.92 | 5.44 ± 0.42 | 0.73 ± 0.03 | 0.28 ± 0.01 | 0.26 ± 0.01 | 0.21 ± 0.01 |
| n(week 1) | 11 | 84 | 11 | 84 | 84 | 84 |
| n(week 2) | 11 | 95 | 11 | 95 | 95 | 95 |
| *p*-value | 0.38623 | 0.34333 | 0.13587 | 0 | 0 | 1.09e-07 |
| Stat. test | T-test | U-test | T-test | T-test2 | U-test | T-test2 |
